# Weighted gene co-expression network reveals driver genes contributing to phenotypes of anaplastic thyroid carcinoma and immune checkpoint identification for therapeutic targets

**DOI:** 10.3389/fonc.2022.1018479

**Published:** 2022-12-01

**Authors:** Xingxing Dong, Yalong Yang, Jinxuan Hou, Weizhen Chen, Qianqian Yuan, Gaoran Xu, Jiuyang Liu, Chengxin Li, Gaosong Wu

**Affiliations:** Department of Thyroid and Breast Surgery, Zhongnan Hospital of Wuhan University, Wuhan, China

**Keywords:** anaplastic thyroid carcinoma, weighted gene co-expression network analysis, enrichment analysis, transcription factor, immune checkpoint, therapeutic target

## Abstract

**Background:**

Anaplastic thyroid carcinoma (ATC) is a rare but extremely malignant tumor, with a rapid growth rate and early metastasis thus leading to poor survival of patients. The molecular mechanisms underlying these aggressive traits of ATC remain unknown, which impedes the substantial progress in treatment to prolong ATC patient survival.

**Methods:**

We applied weighted gene co-expression network analysis (WGCNA) to identify ATC-specific modules. The Metascape web and R package clusterProfiler were employed to perform enrichment analysis. Combined with differentially expressed gene analysis, we screened out the most potential driver genes and validated them using receiver operator characteristic (ROC) analysis, quantitative reverse transcription polymerase chain reaction (qRT-PCR), western blotting, immunohistochemistry (IHC), and triple immunofluorescence staining.

**Results:**

A gene expression matrix covering 75 normal samples, 83 papillary thyroid carcinoma (PTC), 26 follicular thyroid carcinoma (FTC), 19 poor-differentiated thyroid carcinoma (PDTC), and 41 ATC tissue samples were integrated, based on which we detected three most potential ATC-specific modules and found that hub genes of these modules were enriched in distinct biological signals. Hub genes in the turquoise module were mainly enriched in mitotic cell cycle, tube morphogenesis, and cell differentiation, hub genes in the magenta module were mainly clustered in the extracellular matrix organization, positive regulation of cell motility, and regulation of Wnt signaling pathway, while hub genes in the blue module primarily participated in the inflammatory response, innate immune response, and adaptive immune response. We showed that 9 top genes, 8 transcription factors (TFs), and 4 immune checkpoint genes (ICGs) were differentially expressed in ATC compared to other thyroid samples and had high diagnostic values for ATC, among which, 9 novel ATC-specific genes (*ADAM12, RNASE2, CASP5, KIAA1524, E2F7, MYBL1, SRPX2, HAVCR2*, and *TDO2*) were validated with our clinical samples. Furthermore, we illustrated that ADAM12, RNASE2, and HAVCR2 were predominantly present in the cytoplasm.

**Conclusion:**

Our study identified a set of novel ATC-specific genes that were mainly related to cell proliferation, invasion, metastasis, and immunosuppression, which might throw light on molecular mechanisms underlying aggressive phenotypes of ATC and provide promisingly diagnostic biomarkers and therapeutic targets.

## Introduction

Thyroid cancer is the most common endocrine malignancy (1), with a global incidence of 3.0% and mortality of 0.4% (2). It can be divided into three subtypes according to the degree of tumor differentiation: well-differentiated thyroid cancer (WDTC) mainly including papillary thyroid carcinoma (PTC) and follicular thyroid carcinoma (FTC), poor-differentiated thyroid carcinoma (PDTC), and anaplastic thyroid carcinoma (ATC) (3, 4). ATC, deriving from thyroid follicular cells (5), has dramatically different clinical features from other thyroid cancer types: rapid growth rate, early metastasis, and resistance to conventional therapies, thus leading to poor survival of ATC patients (6, 7). The mortality of ATC is the highest (more than 50% of thyroid cancer-related death) (8) and with the 100% disease-specific death (4), though the incidence of ATC is the lowest in thyroid cancer (less than 2%) (9). In recent years, the overall survival (OS) of ATC patients has gradually increased with multimodality therapies including surgery, radiation therapy, and molecular-based personalized treatments (7, 10), but the 2-year OS is still no more than 50% (from 18% to 42%), and the median OS is no more than one year (from 3.16 to 9.5 months) (1, 8).

It was reported that age over 65 years, a history of radiation exposure to the chest or neck, and/or a long-standing goiter were primary risk factors for ATC (5). According to previous studies, ATC tended to affect the elders with age over 60 years (11), especially the eighth decades of life in large studies (6). However, according to the SEER cohort 1986-2015, the incidence of ATC between 35 to 64 years old was 0.72%, while the total incidence was 0.92% (1), implying that these part of ATC patients will not live to the age of 80. Nowadays, immunotherapy is seemingly the most promising treatment for ATC. A study evaluated that PD-1 blockade in ATC had 19% overall response (12) and another study demonstrated that combining an antiangiogenic and antiproliferative tyrosine kinase inhibitor (i.e., Lenvatinib) and an immune checkpoint inhibitor targeting PD-1 was an effective treatment option for ATC with the median progression-free survival of 16.5 months (13). Therefore, it is urgent to find more potential effective targets for ATC. Surely, some researchers have taken their efforts on these, especially with the rise of next-generation sequencing. However, to date, the molecular mechanisms underlying extremely aggressive traits of ATC remain unknown, which impedes the substantial progress in treatment to prolong ATC patient’s survival.

Notably, some studies put their emphasis on gene mutations and found that *TP53* mutation existed in ATC, particularly (3, 14–16). Some researchers paid attention to gene expression alterations. Nevertheless, most of them kept their eyes on ATC and normal thyroid samples (17–19), and/or only focused on fold changes of gene mRNA expression in ATC. For example, Paul Weinberger et al. identified specific differentially expressed genes (DEGs) in ATC compared to normal thyroid and PTC samples and highlighted that cell cycle M-phase genes were up-regulated in ATC (20). Weighted gene co-expression network analysis (WGCNA) is a robust, systems biology approach (21) and has been widely applied in various biological contexts, like autism (22), cancers (23–26), and brain transcriptome atlas (27). WGCNA can be employed to detect clusters (modules) of highly correlated genes, which may reflect true biological signals (e.g., pathways) and can be related to external sample traits. Moreover, the concrete candidate biomarkers or therapeutic targets could be identified based on correlation networks (21).

In the present study, we performed WGCNA based on a gene expression matrix covering 75 normal thyroid tissue samples, 83 PTC, 26 FTC, 19 PDTC, and 41 ATC tissue samples to identify ATC-specific modules that contributed to the devastating features of ATC, then investigated biological processes and pathways reflected by these modules, and further screened out the most potential chief culprits. As a result, we detected the most potential ATC-specific modules confirmed by function and pathway enrichment analyses and found that the notorious characteristics of ATC may largely attribute to dysfunctions in mitotic cell cycle, cell differentiation, blood vessel morphogenesis, cell motility, immune system, and others unknown biological processes beyond our knowledge. Apart from confirming several known ATC-related genes, like *TWIST1*, *SNAI2*, *THSR*, *CTNNB1*, *FOXE1*, and *PAX8*, our study provides a novel set of genes specifically related to ATC, such as *ADAM12*, *RENSE2*, *HAVCR2* and so on, which may deepen our understanding of molecular mechanisms underlying aggressive phenotypes of ATC and provide novel diagnostic biomarkers and therapeutic targets for ATC patients. Furthermore, we identified and validated the most promising driver genes, TFs, and immune checkpoint genes (ICGs) in three ATC-specific modules. Certainly, more *in vivo* and *in vitro* experiments, as well as clinical trials are needed to be accomplished.

## Materials and methods

### Raw data collection

The Gene Expression Omnibus (GEO, https://www.ncbi.nlm.nih.gov/geo/) database is an international public resource to acquire high-throughput gene expression data (28), from which we selected and downloaded 6 gene expression datasets (GSE33630, GSE65144, GSE76039, GSE29265, GSE53157, and GSE82208) of thyroid cancer according to the following two criteria: original CEL file and based on GPL570 platforms ([HG-U133_Plus_2] Affymetrix Human Genome U133 Plus 2.0 Array). In detail, a total of 11 anaplastic thyroid carcinoma (ATC), 49 papillary thyroid carcinoma (PTC), and 45 normal thyroid samples were deposited in the GSE33630 dataset (29), 12 ATC and 13 normal thyroid tissue samples in the GSE65144 dataset (30), 17 poorly differentiated thyroid carcinoma (PDTC) and 20 ATC specimens in the GSE76039 dataset (14), 9 ATC, 20 PTC, and 20 normal thyroid samples in the GSE29265 dataset, 5 PDTC, 15 PTC, 5 follicular thyroid carcinoma (FTC), and 3 normal thyroid samples in the GSE53157 dataset (31), and 27 FTC in the GSE82208 dataset.

### Raw data quality control and preprocessing

Subsequently, R (version 4.0.3) package affyPLM was applied to load probe intensity data stored in CEL files of each dataset respectively and perform regression calculation on the data (32), then the normalized unscaled standard error (NUSE) boxplots were drawn to assess the consistency of samples in each dataset. RNA degradation data were obtained by another R package affy to delineate RNA degradation diagrams, which were used to estimate the degree of RAN degradation in each gene chip (i.e., sample). Rules as a thumb, specimens with a median standard error higher than 1.05 were excluded because the consistency between them and others in the same dataset was considered to be poor. Samples whose RNA degradation curves showed much higher slopes and poor uniformity with others were also considered to be eliminated to guarantee data quality at the probe level (33).

After deleting deficient gene chips, we reintegrated raw CEL files by tissue types (i.e., normal thyroid samples, PTC, FTC, PDTC, and ATC samples), then carried out the log scale robust multi-array analysis (34) to turn probe intensity data into expression values through affy package in R with three main steps: background correction, normalization and log2 transformation of PM values, converted probe IDs to gene symbols with platform annotation file (i.e., GPL570-55999.txt), deleted rows that didn’t correspond to any gene symbols in excel files and finally carried out K Nearest Neighbors (KNN) algorithm (35) to estimate and impute the missing value of gene expression matrix by using impute package of R.

Furthermore, the principal component analysis (PCA) was applied to estimate the degree of similarity among thyroid normal and various cancer tissues, performed by the Sangerbox tools, a free online platform for data analysis (http://www.sangerbox.com/tool). The samples that were outliers were deleted.

### Weighted gene co-expression network analysis

Weighted gene co-expression network analysis (WGCNA) has been widely employed to explore the system-level function of genes, by which the gene co-expression network constructed with the scale-free topology criterion could provide functional explanations of system biology (36). The corresponding R package WGCNA (version 1.70-3) was applied to perform WGCNA based on the top 20% of most variant genes, which mainly included the following steps: (1) construct a weighted gene co-expression network and detect modules. Here, we chose the soft power adjacency function parameter β (i.e., soft threshold) according to the scale-free topology criterion: signed R2 > 0.80, high mean connectivity, and the regression line between log(p(k)) and log(k) around -1, which were depicted by network properties plots and scale-free topology plots. The topological overlap matrix -based dissimilarity combined with the hierarchical clustering method was applied to identify modules as described in (36); (2) identify ATC-specific modules. In the present study, we integrated microarray data of six datasets covering normal thyroid tissues, PTC, FTC, PDTC, and ATC tissues to detect modules that were only significantly positive or negative correlated to ATC (R > 0.5, p < 0.05); (3) reconfirm the ATC-specific modules and find key drivers from them. We further extracted module eigengenes (MEs, the first principal components of a module) from every module, calculated gene significance (GS) of every gene and each module (The average absolute GS (aGS) of all genes in one module denoted module significance), and evaluated the correlations between GS and module membership (MM). In modules related to ATC, the MEs with a high absolute value of GS also have high MM values. Therefore, we selected genes with aGS > 0.2 and the corresponding p < 0.05 as hub genes for the further function and pathway enrichment analyses; (4) export gene co-expression network files (i.e., edge.txt and node.txt) for visualization and further analysis.

### Enrichment analysis and TP53 mutation analysis

Metascape web (http://metascape.org) was employed to perform enrichment analysis, a user-friendly portal, and combined functional enrichment, interactome analysis, gene annotation, and membership search to leverage over 40 independent knowledgebases (37). We put gene lists of interest into Metascape, chose “H. sapiens” for “put as species” and “analysis as species”, and clicked “Express Analysis”, then the enrichment results were automatically finished with p < 0.05 as the default setting. Metascape incorporates the biological process (BP) category of Gene Ontology (GO), Kyoto Encyclopedia of Genes and Genomes (KEGG), Reactome gene sets, canonical pathway, and CORUM complexes databases. GO is a strong gene ontology resource widely used in gene functional analysis, composed of BP, molecular function (MF), and cellular component (38). KEGG is an integrated database for biological interpretation of genome sequences and other high-throughput data, where KEGG pathway mapping is available to any cellular organism (39). The CC and MF categories of GO enrichment analysis were accomplished by R package clusterProfiler (version 3.16.1) with an adjusted p-value Cutoff = 0.05. Meanwhile, the KEGG pathways involved were also evaluated by R package clusterProfiler with an adjusted p-value Cutoff = 0.05.

The TP53 mutation analysis on thyroid cancer was performed by the cBioPortal database (40) (http://www.cbioportal.org/) based on two studies (MSKCC and TCGA, Firehose Legacy), which contained a total of 633 thyroid cancer samples, including 33 ATC samples.

### TF, immune checkpoint exploration and differentially expressed gene identification

Transcription factor information was downloaded from Transcriptional Regulatory Relationships Unraveled by Sentence-based Text mining 35 (TRRUST; www.grnpedia.org/trrust), which consisted of 800 TFs in humans (41). Forty-nine immune checkpoint genes (ICGs) were collected referring to (42).

We integrated data of GSE33630, GSE65144, and GSE76039 (due to their more ATC samples) into two gene expression matrices (ATC versus normal and ATC versus PTC) and carried out the ComBat function in R package sva to remove batch effects. Differentially expressed genes (DEGs) were identified by linear models of Microarrays (limma) (43) package of R with a criterion of log2(Fold Change)>1 or log2(Fold Change) < -1 and adjust P value <0.05. The overlapping genes were obtained by Venn diagrams using online tools (http://bioinformatics.psb.ugent.be/webtools/Venn/).

### Diagnostic value analysis

The receiver operating characteristic (ROC) curve and the corresponding area under the curve (AUC) were applied to evaluate the diagnostic value of candidate genes for ATC and were carried out through the Sangerbox tools mentioned above. We took “non-ATC” (i.e., all other samples except for ATC samples) and “ATC” as “response”, and used the corresponding gene expression value as “predictor”. Those genes with AUC > 0.9 were selected for further validation.

### Thyroid cancer sample collection

Twelve paired PTC and one ATC samples were obtained from patients who underwent thyroid surgery at Zhongnan Hospital of Wuhan University with the informed consent of the patients. Tissues were divided into two parts, one instantly frozen by liquid nitrogen and then stored at −80°C for total RNA and protein extraction, the other fixed with formalin and embedded in paraffin for immunohistochemical and immunofluorescent analyses. The usage of human specimens was approved by the Ethics Committee of Zhongnan Hospital of Wuhan University.

### Quantitative reverse transcription polymerase chain reaction and western blotting analysis

Total RNA and protein were extracted from the tissues using DNA/RNA/protein isolation kits (TIANGEN, China) according to the manufacturer’s instructions. The RNA samples were converted to complementary DNA (cDNA) *via* the ABScript III RT Master Mix (ABclonal) for the following qRT-PCR that was implemented with 2X Universal SYBR Green Fast qPCR Mix (ABclonal) in triplicate. The 2^−ΔΔCT^ method was used to normalize each gene with *GAPDH* as the internal control and the average of ΔCT value of normal thyroid samples as the baseline control. The primer sequences for qRT - PCR used in the study were designed and selected from the Primer designing tool (http://www.ncbi.nlm.nih.gov/tools/primer-blast/), and listed in **Supplementary Table 1**. The WB was implemented to evaluate the protein expression level according to standard procedure. The gray density of bands was measured by Image J software. GAPDH was used as the internal control and the normalized average of normal thyroid samples as the baseline control. The mouse monoclonal antibody against GAPDH was purchased from proteintech (China). The rabbit polyclonal antibodies against ADAM12 (#A7940), RNASE2 (#A9949), KIAA1524 (#A12267), HAVCR2 (#A2516), E2F7 (#A15211), and MYBL1 (#A9829) were obtained from ABclonal (China). The secondary antibodies (Goat anti-rabbit and Goat anti-mouse IgG) were obtained from Solarbio (China). The dilutions of antibodies used in this study were presented in **Supplementary Table 2**.

### Immunohistochemistry and triple immunofluorescence staining

Paraffin-embedded tissues were cut into about 4-micron-thick sections and then processed to be blocked with 3% BSA according to standard procedures. Subsequently, for immunohistochemistry, the slides were incubated with primary antibodies (1:100, ADAM12, HAVCR2, and RNASE2) mentioned above, followed by incubation with an HRP-conjugated anti-rabbit secondary antibody (ab205718, abcam, UK). For triple immunofluorescence, the slides were first incubated overnight at 4 °C with rabbit polyclonal antibody against ADAM12 (1:200). The next day, the slides were labeled with CY3 conjugated anti-rabbit lgG (1:500, ab6939, abcam, UK) for 50 min at room temperature. After being rinsed with TBST for 15 min, the slides were incubated overnight at 4 °C with rabbit polyclonal antibody against HAVCR2 (1:200). On the third day, the slides were labeled with 488 conjugated anti-rabbit lgG (1:500, ab150077, abcam) for 50 min and were subsequently incubated overnight at 4 °C with rabbit polyclonal antibody against RNASE2 (1:200). On the fourth day, the slides were labeled with CY5 conjugated anti-rabbit lgG (1:500, ab6564, abcam, UK) for 50 min at room temperature. Then, the slides were scanned with scanister (Pannoramic MIDI, 3DHISTECH Ltd, Hungary) and observed using CaseViewer software.

### Key gene co-expression network visualization and co-expressed gene enrichment analysis

Cytoscape (version 3.9.1), a useful software to visualize biological molecular interaction networks (44), was applied to visualize key gene co-expression networks with network TXT files of edges acquired by WGCNA. Enrichment analysis of co-expressed genes was accomplished by Metascape web.

### Statistical analysis

In this study, the statistical analyses accomplished by R software and Metascape web were automatically calculated with an adjusted p-value < 0.05 or p-value < 0.01 considered statistically significant. Data of qRT-PCR are presented as the means ± standard error of the mean, while data of WB are displayed by boxplots. Statistical analyses were performed by GraphPad Prism version 8.0.1 using unpaired one-way analysis of variance (ANOVA) with Tukey’s test.

## Results

### Quality controlled and pre-processed data

After deleting samples with poor consistency with others in the same dataset, the median standard errors of the rest of the specimens shown in the NUSE boxplots (**Supplementary Figures 1A–F**. left) are close to 1 and no more than 1.05, which indicates good agreement among them, and the RNA degradation plots of them exhibited in **Supplementary Figures 1A–F** (right) are considered to be acceptable. As a result, a total of 10 ATC, 48 PTC, and 42 normal thyroid tissue samples from the GSE33630 dataset, 11 ATC and 10 normal thyroid tissue samples from the GSE65144 dataset, 19 ATC and 16 PDTC tissue samples from the GSE76039 dataset, 8 ATC, 20 PTC, and 20 normal thyroid tissue samples from the GSE29265 dataset, 4 FTC, 15 PTC, 4 PDTC, and 3 normal thyroid tissue samples from the GSE53157 dataset, and 22 FTC tissue samples from the GSE82208 dataset were obtained, which, to some extent, had higher quality at probe level and were more credible. Then through data standardization, transformation and probe set annotation, gene expression matrices concluding a total of 20460 genes were acquired. For WGCNA, all these samples were used and finally we integrated 75 normal thyroid tissue samples, 83 PTC, 26 FTC, 19 PDTC, and 41 ATC tissue samples into one expression matrix, the PCA diagram of which displayed that ATC samples were clustered separately from other types of thyroid cancar (**Supplementary Figure 2A**). For DEG identification, we merged GSE33630, GSE65144, and GSE76039 datasets (they contained more ATC samples than others) into two expression matrices and removed batch effects. One included 40 ATC and 52 normal thyroid tissue samples, the other consisted of 40 ATC and 48 PTC tissue samples.

### Weighted gene co-expression network and ATC-specific modules

**Figure 1:**
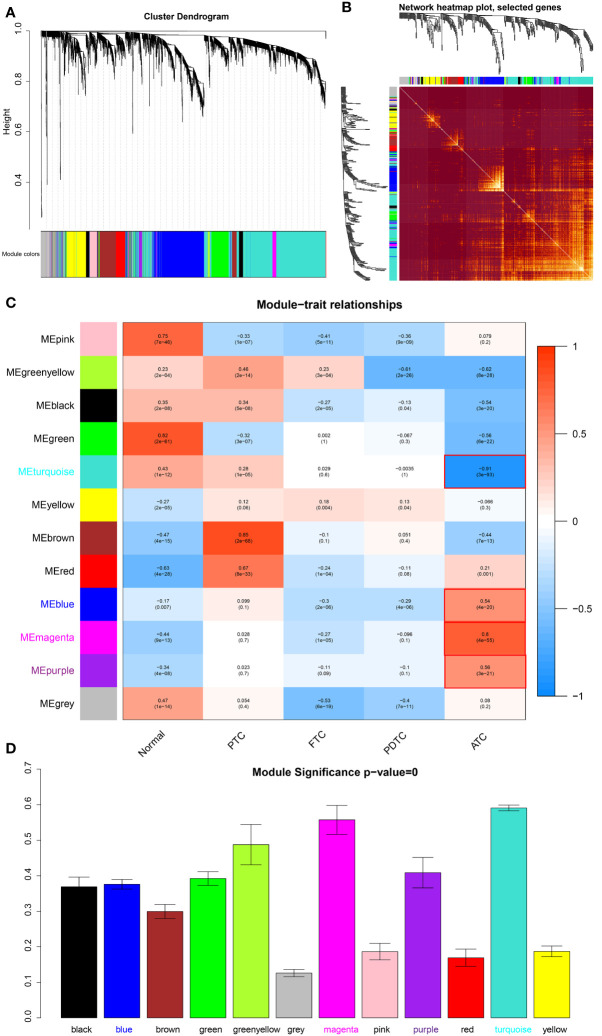
Module detection and ATC-specific module identification. **(A)** Hierarchical clustering dendrogram of the top 20% variant genes. The color row underneath the dendrogram displays the corresponding module assignment determined by the Dynamic Tree Cut. One color represents one module. **(B)** Heatmap diagram of topological overlap in the gene network drawn based on 400 selected genes. Each row and column correspond to a gene, dark red color represents low topological overlap, and a gradually lighter color denotes higher topological overlap. Lighter squares along the diagonal correspond to modules. The gene clustering dendrogram and module assignment are exhibited along the left and top. **(C)** Heatmap of the correlations between module eigengenes and various subtypes of thyroid cancer samples, including normal thyroid samples. The table is color-coded by correlation based on the color legend, blue color represents negative correlation, while red color represents positive correlation. The number in the rectangle is the concrete correlation coefficient and p-value (in the bracket). Modules marked in red are those that are significantly positively or negatively correlated only with ATC. **(D)** Barplot of mean ATC-based GS across modules (module significance). The higher the mean GS in a module, the more significantly correlated the module is to ATC. Normal, normal thyroid tissue samples; PTC, papillary thyroid carcinoma; FTC, follicular thyroid carcinoma; PDTC, poor-differentiated thyroid carcinoma; ATC, anaplastic thyroid carcinoma.

We screened out the top 20% of most variant genes (4092 out of 20460) based on a gene expression matrix that covered 75 normal thyroid tissue samples, 83 PTC, 26 FTC, 19 PDTC, and 41 ATC tissue samples to construct the weighted gene co-expression network. The cluster tree of these samples shown in **Supplementary Figure 2B** reveals that ATC samples are clustered together and separate from other samples. According to the scale-free topology criterion and consideration of mean connectivity, we chose soft threshold power b = 7, with which the R^2 =^ 0.83 > 0.80 and the mean connectivity was not too low (**Supplementary Figures 2C, D**), indicating that the network constructed satisfied the scale-free topology approximately and included proper information. Based on the network, 11 modules shown in **Figure 1A** represented by different colors are significantly identified, excluding the grey module that contained genes without significant enrichment in any modules. Moreover, the TOM plot of 400 randomly selected genes (**Figure 1B**) shows that the connections among them are tight in these modules, especially in yellow, brown, blue, and turquoise modules.

In order to identify modules that play an important role in ATC, we took the main pathological types of thyroid cancer as clinical traits, thus which modules that were uniquely significantly correlated to ATC were explicit. As shown in **Figure 1C**, the turquoise module is significantly negative associated with ATC (R = -0.91 and p = 3e-93), while the blue (R = 0.54, p = 4e-20), magenta (R = 0.8, p = 4e-55), and purple (R = 0.56, p = 3e-21) modules have significantly positive correlations to ATC, indicating that genes of these modules may be more potential to contribute to the phenotypes of ATC. Moreover, the hierarchical clustering dendrogram of eigengenes and the heatmap diagram of the adjacencies in the eigengene network also revealed a negative correlation between the turquoise module and ATC and positive correlations of blue, magenta, and purple modules with ATC (**Supplementary Figures 3A, B**). Correspondingly, the eigengene expression level of the turquoise module showed an overall downward trend in ATC samples and an upward trend in normal thyroid tissue samples, PTC, FTC, and PDTC samples (**Supplementary Figure 3C**), while eigengene expression levels of blue, magenta and purple modules were generally up-regulated in ATC and down-regulated in other samples (**Supplementary Figures 3D–F**), suggesting that the majority of genes of the turquoise module were low expressed in ATC and many genes of blue, magenta and purple modules were highly expressed. Additionally, to further determine the significance of these four modules to ATC, as well as eigengenes of them, GS was measured for every gene and the average absolute GS (aGS) of all genes in one module denoted module significance. As shown in **Figure 1D**, module significance values of these four modules are over 0.3 with p = 0. Meanwhile, the GS for ATC exhibited very significant associations with MM in these four modules: cor = 0.93, p < 1e-200 in turquoise module, cor = 0.51, p = 3.5e-54 in blue module, cor = 0.92, p = 5.1e-40 in magenta module, and cor = 0.68, p = 2e-07 in purple module (**Supplementary Figures 3G–J**), revealing that hub genes of these four modules also tend to be highly correlated to ATC. These results suggested that genes in these four modules may be more biologically significant to ATC. Hence, we extracted hub genes from them (aGS > 0.2, p < 0.05) for further function and pathway enrichment analyses. In detail, a total of 1548 hub genes were acquired from the turquoise module, 628 hub genes from the blue module, 96 hub genes from the magenta module, and 41 hub genes from the purple module.

### Crucial function and pathway disturbances in ATC

**Figure 2:**
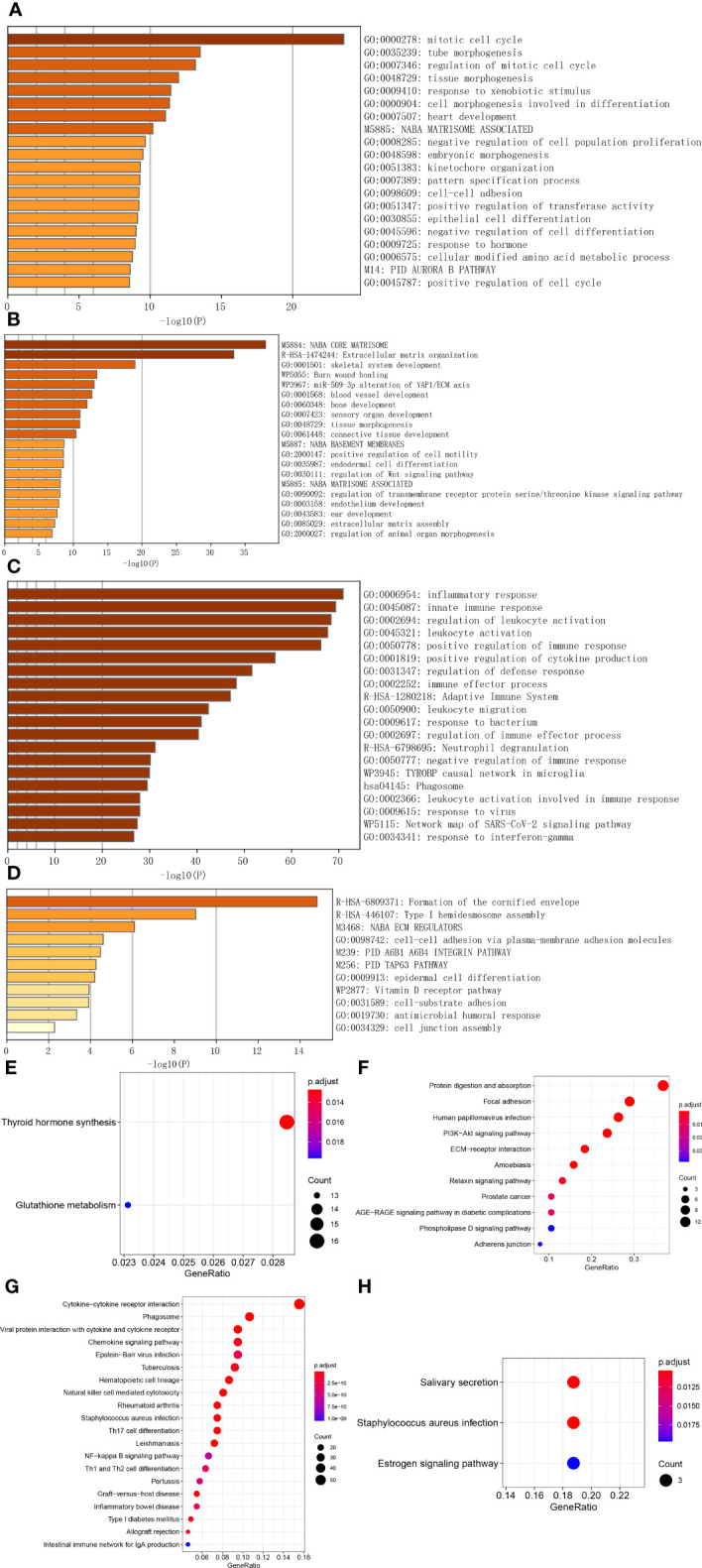
Function and pathway analyses of hub genes in ATC-specific modules. **(A–C)** The top 20 biological signals or pathways significantly enriched by hub genes of turquoise, magenta, and blue module, respectively. **(D)** All biological signals or pathways significantly enriched by the hub genes of the purple module. **(E–H)** KEGG pathways significantly enriched by hub genes of turquoise, magenta, blue, and purple module, respectively, among which, the top 20 pathways were shown for the blue module.

Through WGCNA, four ATC-specific modules were identified, as well as hub genes in them. To test whether these modules reflect true biological processes and to detect which vital perturbations occurred to ATC. We performed function and pathway enrichment analyses for hub genes. As a result, the hub genes from turquoise module were significantly enriched (p < 0.01) in a total of 281 main biological processes or pathways (**Figure 2A** and **Supplementary Table 3**) and the top five of which were “mitotic cell cycle” (95 genes, LogP = -23.58), “tube morphogenesis” (82 genes, LogP = -13.50), “regulation of mitotic cell cycle” (66 genes, LogP = -13.16), “tissue morphogenesis” (70 genes, LogP = -11.98), and “response to xenobiotic stimulus” (56 genes, LogP = -11.43). The hub genes from magenta module were significantly clustered in 43 main biological processes or pathways (**Figure 2B** and **Supplementary Table 4**) and the top five of which were “NABA core matrisome” (30 genes, LogP = -37.97), “extracellular matrix organization” (28 genes, LogP = -33.34), “skeletal system development” (22 genes, LogP = -18.98), “Burn wound healing” (11 genes, LogP = -13.43), and “miR-509-3p alteration of YAP1/ECM axis” (7 genes, LogP = -13.03). For blue module, the hub genes from it were significantly gathered in a total of 287 main biological processes or pathways (**Figure 2C** and **Supplementary Table 5**) and the top five of which were “inflammatory response” (103 genes, LogP = -70.90), “innate immune response” (120 genes, LogP = -69.29), “regulation of leukocyte activation” (110 genes, LogP = -68.33), “leukocyte activation” (101 genes, LogP = -67.62), and “positive regulation of immune response” (103 genes, LogP = -66.17). As for the purple module, the hub genes from it were significantly enriched in 11 main biological processes or pathways (**Figure 2D** and **Supplementary Table 6**), the top three of which were “formation of the cornified envelope” (10 genes, LogP = -14.81), “type I hemidesmosome assembly” (4 genes, LogP = -9.02), “NABA ECM regulators” (6 genes, LogP = -6.10). Apart from these, we noted that 96 hub genes in blue module were significantly enriched in “immunosuppression” (LogP = -54) according to DisGeNET (**Supplementary Table 7**) and based on PaGenBase, 131, 102, and 47 hub genes in blue module were significantly enriched in spleen (LogP = -100), blood (LogP = -100), and thymus (LogP = -34), respectively (**Supplementary Table 7**). Moreover, 29 hub genes of the turquoise module, 4 hub genes of the magenta module, and 14 hub genes of the blue module were discerned to be regulated by TP53 (**Supplementary Table 7**). Based on the cBioPortal database, we found that *TP53* mutation was present in more than 60% of ATC patients much more than in other thyroid cancer patients (PTC, FTC, and PDTC), and significantly associated with the overall survival of patients with thyroid cancer (p = 0) (**Supplementary Figure 4**).

In the meantime, we also applied the R package to implement KEGG pathway enrichment analysis and found that 2 pathways were significantly enriched by turquoise module genes, which were “thyroid hormones synthesis” and “glutathione metabolism” (**Figure 2E** and **Supplementary Table 8**), 11 pathways were enriched by magenta module genes, such as “protein digestion and absorption”, “focal adhesion”, “human papillomavirus infection”, “PI3K-Akt signaling pathway”, “ECM-receptor interaction”, etc. (**Figure 2F** and **Supplementary Table 8**), a total of 64 pathways were enriched by blue module genes (**Supplementary Table 8**), the top 20 of which were shown in **Figure 2G**, including “cytokine-cytokine receptor interaction”, “phagosome”, “viral protein interaction with cytokine and cytokine receptor”, “chemokine signaling pathway”, “NF-kappa B signaling pathway”, “Th1 and Th2 cell differentiation” and so on. For purple module genes, 3 pathways were significantly enriched by them, they were “salivary secretion”, “staphylococcus aureus infection”, and “estrogen signaling pathway” (**Figure 2H** and **Supplementary Table 8**).

Additionally, we investigated the subcellular localization and molecular function of these hub genes by applying CC and MF categories of GO. As listed in **Supplementary Table 9**, the turquoise module hub genes are significantly enriched in a total of 37 CC terms, the top three of which are “condensed chromosome, centromeric region” (28 genes, p. adjust = 3.49E-07), “kinetochore” (30 genes, p. adjust = 3.49E-07), and “chromosome, centromeric region” (37 genes, p. adjust = 3.49E-07); The magenta module hub genes are significantly enriched in 13 CC terms, the top three of which are “collagen-containing extracellular matrix” (37 genes, p. adjust = 8.27E-38), “collagen trimer” (16 genes, p. adjust = 2.35E-20), and “endoplasmic reticulum lumen” (22 genes, p. adjust = 2.54E-19); The blue module hub genes are significantly enriched in a total of 58 CC terms, the top three of which are “external side of plasma membrane” (58 genes, p. adjust = 4.06E-22), “secretory granule membrane” (42 genes, p. adjust = 3.18E-15), and “MHC protein complex” (12 genes, p. adjust = 1.51E-10); The purple module hub genes are significantly enriched in 8 CC terms, the top three of which are “intermediate filament” (6 genes, p. adjust = 0.0002), “cornified envelope” (4 genes, p. adjust = 0.0002), and “intermediate filament cytoskeleton” (6 genes, p. adjust = 0.0002). While the MF terms enriched are listed in **Supplementary Table 10**, the turquoise module hub genes are significantly enriched in two MF terms, which are “cell-cell adhesion mediator activity” (15 genes, p. adjust = 0.0007) and “cell adhesion mediator activity” (16 genes, p. adjust = 0.0007); The magenta module hub genes are significantly enriched in 22 MF terms, the top three of which are “extracellular matrix structural constituent” (29 genes, p. adjust = 1.64E-36), “extracellular matrix structural constituent conferring tensile strength” (14 genes, p. adjust = 2.37E-21), and “platelet-derived growth factor binding” (7 genes, p. adjust = 7.90E-13); The blue module hub genes are significantly enriched in a total of 62 MF terms, the top three of which are “immune receptor activity” (38 genes, p. adjust = 2.76E-24), “cytokine receptor activity” (27 genes, p. adjust = 2.05E-16), and “cytokine binding” (28 genes, p. adjust = 4.19E-14); The purple module hub genes are significantly enriched in 7 MF terms, the top three of which are “structural constituent of cytoskeleton” (5 genes, p. adjust = 0.0002), “endopeptidase inhibitor activity” (5 genes, p. adjust = 0.0009), and “peptidase inhibitor activity” (5 genes, p. adjust = 0.0009).

These results indicated that the co-expression modules detected were biologically meaningful and uncovered that mitotic cell cycle, cell differentiation, blood vessel morphogenesis, cell motility, immune system, or others beyond our knowledge were responsible for fatal traits of ATC, especially turquoise, magenta, and blue modules. Therefore, we decided to further validate potential key drivers of ATC from these three modules.

### Potential key drivers in ATC

**Figure 3:**
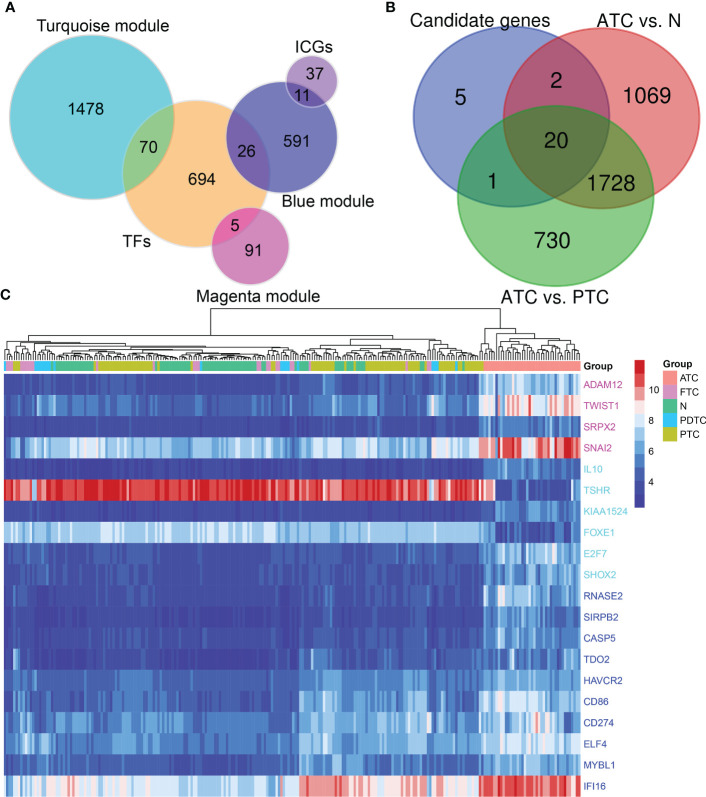
Potential key drivers screening from ATC-specific modules. **(A)** TF and immune checkpoint gene detection. **(B)** Candidate genes are the top 3 genes and TFs with the highest aGS in turquoise, magenta, and blue modules, and ICGs in the blue module. Combined with differentially expressed genes in ATC compared to normal thyroid tissue samples and PTC to identify 20 key genes. **(C)** The hierarchical clustering heatmap shows expression levels of these 20 hub genes across all samples and gathers ATC samples together. TF, transcription factor; ICG, immune checkpoint gene; N, normal thyroid samples.

**Figure 4:**
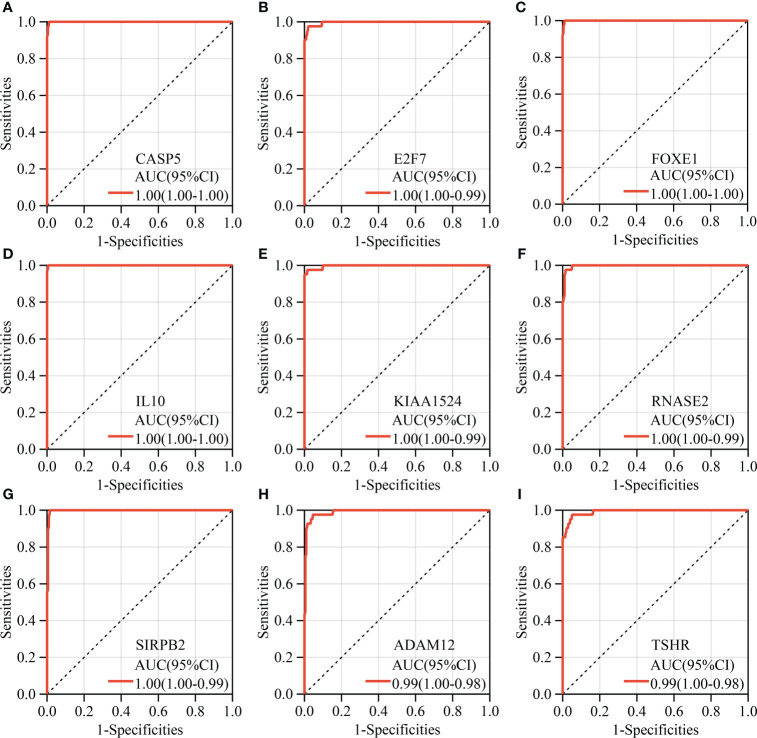
Diagnostic ROC analysis of hub genes. **(A–I)** The top nine key genes with the largest AUC of ROC.

A total of 2272 hub genes in turquoise, magenta and blue modules are plentiful, as well as biological processes and pathways enriched by them, thus we just attempt to focus on the most potential, functional, and specific ones in ATC. To our knowledge, TF, with global functions, can regulate the transcription of other genes by binding to specific DNA sequences and are relevant to many diseases and phenotypes (45). Immune checkpoints have promising therapeutic prospects (12, 46, 47). Therefore, we took TFs and immune checkpoints into account apart from the top 3 hub genes with the highest aGS in each module. Consequently, we found 70 TFs from the turquoise module hub genes, 5 TFs from the magenta module, 26 TFs from the blue module, and 11 immune checkpoint genes from the blue module (**Figure 3A**). In addition to novel TFs and ICGs in ATC, we noted that several genes identified had been widely accepted in ATC and even had been applied in the clinic, like *CTNNB1*, *PAX8*, *FOXE1*, and *CD274* (PD-L1) (12, 48–50) (**Supplementary Table 11**). Under the consideration that different module reflects different biological signals and too many TFs, we finally selected the top 3 genes and the top 3 TFs with the highest aGS in each module and 11 immune checkpoint genes (ICGs) as candidate genes. Combined with DEGs, we discovered that a total of 20 candidate genes (9 top genes *ADAM12, TWIST1, SRPX2, RNASE2, SIRPB2, CASP5, IL10, TSHR*, and *KIAA1524*), 8 top TFs *TWIST1, SNAI2, ELF4, IFI16, MYBL1, FOXE1, E2F7*, and *SHOX2*, and 4 ICGs (*CD86, CD274, HAVCR2*, and *TDO2*) were differentially expressed in ATC tissue samples compared to normal thyroid tissue samples and PTC tissue samples, of which 18 genes (*ADAM12, TWIST1, SRPX2, RNASE2, SIRPB2, CASP5, IL10, KIAA1524, SNAI2, ELF4, IFI16, MYBL1, FOXE1, E2F7*, *CD86, CD274, HAVCR2*, and *TDO2)* were up-regulated in ATC while 2 genes (*FOXE1* and *TSHR*) were down-regulated (**Figure 3B** and **Supplementary Table 12**). In addition, based on the expression profile of these 20 genes, ATC samples were successfully clustered together (**Figure 3C**). More convincingly, AUCs of ROC curves of these 20 genes were all over 0.9 except for *CD274* (**Figure 4** and **Supplementary Figures 5A–K**), suggesting that these genes were specific to ATC and could distinguish it from other types of thyroid cancer as ATC-specific markers like *CDH1* (AUC = 0.99) and *PAX8* (AUC = 0.97) (**Supplementary Figures 5L, M**), and maybe even have more diagnostic value than some other existed markers of ATC, such as *CTNNB1* (AUC = 0.76) and *TTF1* (AUC = 0.58) (**Supplementary Figures 5N, O**) at the mRNA expression level.

### Key gene validation in clinical samples

**Figure 5:**
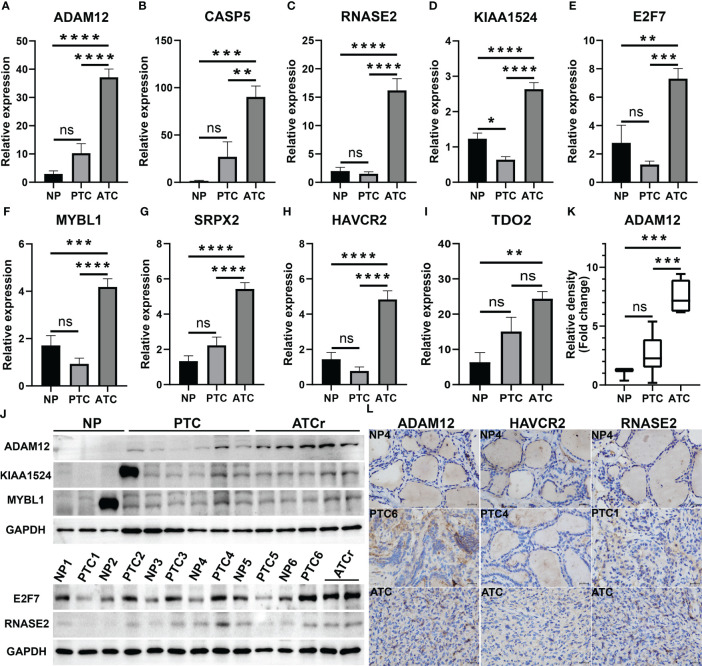
Validation of novel ATC-specific key genes. **(A–I)** The mRNA expression levels of nine key genes in one ATC sample compared to PTC and paired normal thyroid samples evaluated using qRT-PCR. Among them, *E2F7* and *MYBL1* are TFs, *HAVCR2* and *TDO2* are ICGs. **(J, K)** Protein expression levels of top four driver genes in clinical samples detected by western blotting (WB) and the corresponding semi-quantitative analysis of ADAM12. **(L)** Representative immunohistochemistry (IHC) staining for ADAM12, HAVCR2, and RNASE2 expression in clinical samples (*X* 400). **p < 0.01, ***p < 0.001, ****p < 0.0001. NP, PTC paired normal thyroid tissues; NP1-6, normal thyroid tissues from PTC patients numbered 1-6; PTC1-6, PTC tissues from PTC patients numbered 1-6; ATCr, ATC tissues from one ATC patient; ns, no significance.

**Figure 6:**
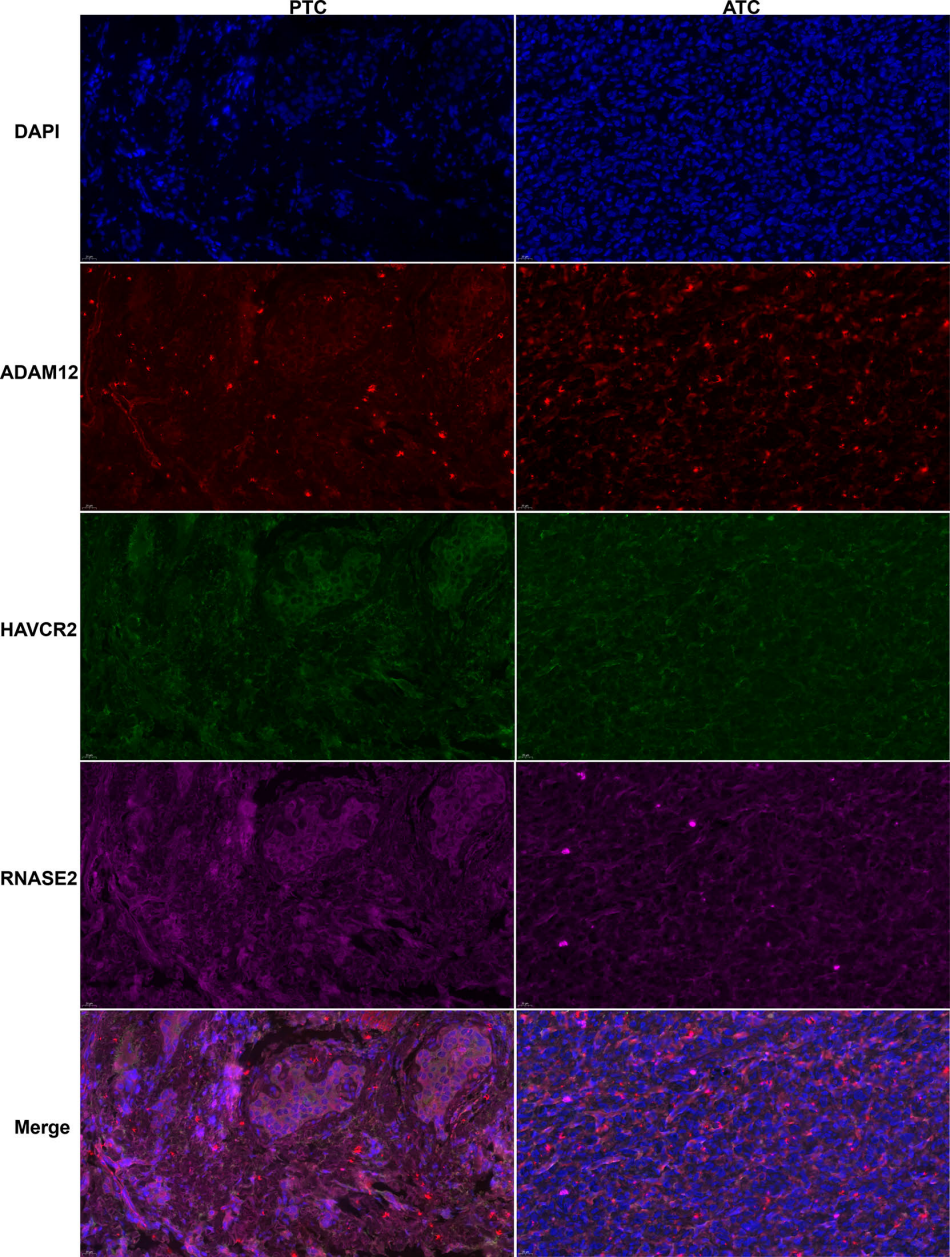
Typical triple immunofluorescence staining for ADAM12 (red), HAVCR2(green), and RNASE2 (purple) localization in PTC and ATC samples (*X* 400).

Among these 20 key genes, five genes *TWIST1* (45), *SNAI2* (46), *TSHR* (47), *FOXE1* (48), and *CD274* (PD-L1) (49) have been reported to be differentially expressed in ATC compared to normal thyroid samples and other thyroid cancer samples both at mRNA and protein expression levels. Therefore, we further validated the other 15 key genes at the mRNA level using our clinical samples (12 paired normal and PTC samples and one ATC sample). The results showed that a total of nine key genes *ADAM12*, *CASP5*, *RNASE2*, *KIAA1524*, *E2F7*, *MYBL1*, *SRPX2, HAVCR2*, and *TDO2* were significantly up-regulated in this one ATC sample compared to PTC and/or paired normal thyroid samples (p < 0.01, **Figures 5A–H**), among which *E2F7* and *MYBL1* were TFs, and *HAVCR2* and *TDO2* were ICGs. While other key genes had no or less significant changes in this one ATC sample compared to PTC or paired normal thyroid samples (p > 0.05 or p < 0.05, **Supplementary Figure 6A**). Consequently, we selected the top significantly up-regulated key gene and TF obtained from different modules (*ADAM12* from the magenta module, *KIAA1524* and *E2F7* from the turquoise module, and *RNASE2* and *MYBL1* from the blue module), and the top up-regulated ICG, HAVCR2 for their protein expression verification through the WB experiment. As shown in **Figure 5J** and **Supplementary Figures 6B–F**, all target proteins had expressions in this one ATC sample (at least two duplicates), while in PTC and paired normal thyroid tissue samples, their expression levels varied. However, with our available protein samples of clinical tissues, we found only one protein ADAM12 was significantly up-regulated in this one ATC sample compared to PTC and paired normal thyroid samples (p < 0.001, **Figure 5K**), while other proteins had no significant overexpression (**Supplementary Figure 6G**) and the expression of one protein HAVCR2 was hard to be quantitatively evaluated because the objective and nonspecific bands of it were difficult to be separated (**Supplementary Figure 6D**). Meanwhile, the protein expression levels of ADAM12, HAVCR2, and RNASE2 were assessed using IHC. As shown in **Figure 5L** and **Supplementary Figure 7**), all of them had positive expressions in this one ATC sample. However, the results of IHC were hard to be semi-quantitatively analyzed due to too much nonspecific staining in PTC and paired normal thyroid tissue samples. Furthermore, we performed triple immunofluorescence staining for ADAM12, HAVCR2, and RNASE2 to further investigate their locations in PTC and ATC cells. As shown in **Figure 6**, these three proteins were almost located in the same cells in the same tissue microenvironment and all of them were predominantly present in the cytoplasm.

### Key gene co-expression network and biological signals involved

**Figure 7:**
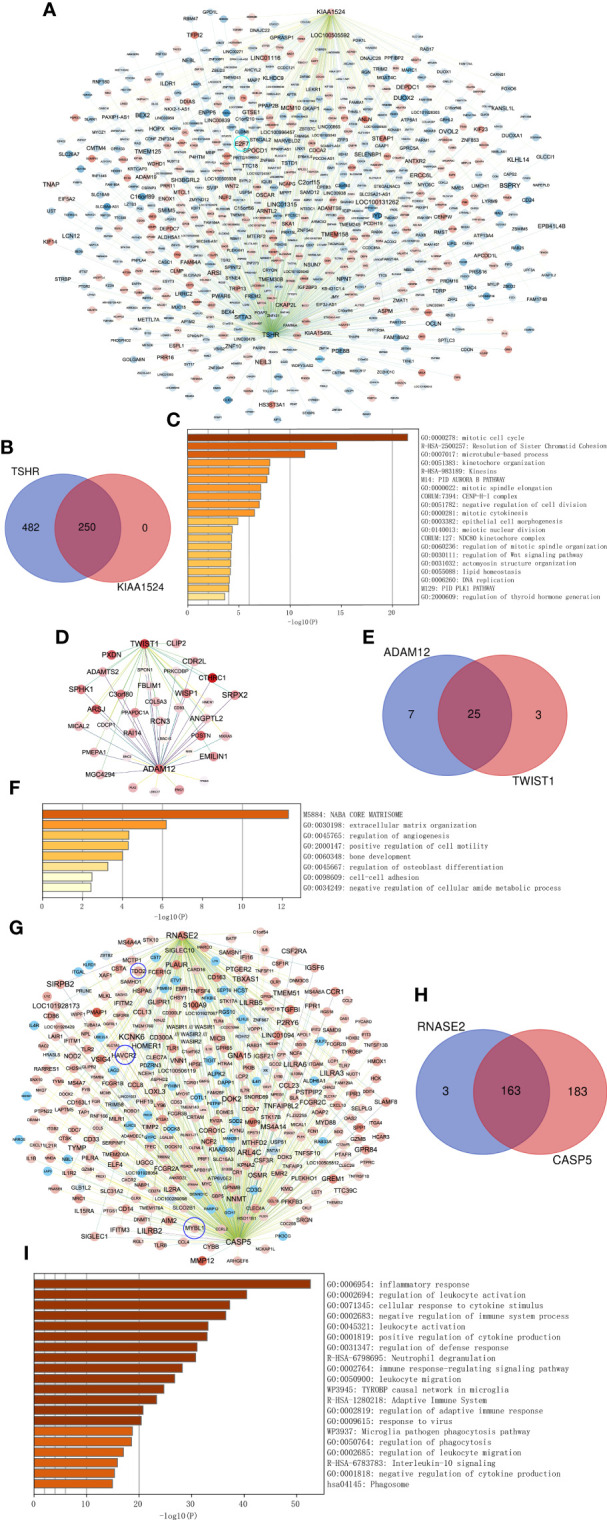
Co-expressed networks of two top genes and enrichment analysis. **(A)** Co-expressed hub genes of *TSHR* and *KIAA1524* in the turquoise module. Each node represents a gene. The blue nodes denote lower expressed genes in ATC compared to normal thyroid samples, while the red nodes denote higher expressed genes. The darker the node, the more differentially expressed the gene between ATC and normal thyroid samples. The edges between nodes indicate co-expression between them. The darker the edges, the stronger the co-expression relationship. **(B)** Overlapping co-expressed genes of *TSHR* and *KIAA1524*. **(C)** Top 20 Biological processes and pathways enriched by all co-expressed genes of *TSHR* and *KIAA1524*. **(D–F)** The same as A-C, but in the magenta module and two key genes *ADAM12* and *TWIST1* in this module. **(G–I)** The same as A-C, but in the blue module and two key genes *RNASE2* and *CASP5* in this module.

Combined with existing studies and our validation results, we found that key genes *TSHR* and *KIAA1525* from the turquoise module, *ADAM12* and *TWIST1* from the magenta module, and *RNASE2* and *CASP5* from the blue module with higher aGS for ATC may play more crucial roles in ATC. Therefore, we extracted co-expressed genes of them and performed enrichment analysis on these genes to further prove their pivotal roles in ATC and to investigate functions and pathways they may participate in. We selected co-expressed genes having a weighting coefficient over 0.1 with the key gene for the turquoise module while over 0.05 for magenta and blue modules to visualize networks and to explore functions and pathways involved because genes in the turquoise module were too many. In consequence, *TSHR* had a total of 732 co-expressed genes, of which 250 were *KIAA1524* co-expressed genes, including *E2F7* (**Figures 7A, B**). These genes were mainly enriched in “mitotic cell cycle”, “Resolution of Sister Chromatid Cohesion”, “microtubule-based process” and so on (**Figure 7C**), indicating that *TSHR* and *KIAA1524* may be regulated by E2F7 and promote AT*C* though these biological processes and pathways*. ADAM12* had 32 co-expressed genes, while *TWIST1* had 28, of which 25 were overlapped (**Figures 7D, E**). All of these genes primarily clustered in “NABA CORE MATRISOM”, “extracellular matrix organization”, “regulation of angiogenesis”, etc. (**Figure 7F**), and *ADAM12* was co-expressed with *TWIST1* (**Figure 7D**), suggesting that *ADAM12* may be regulated by TWIST1 and contribute to ATC by these biological processes and pathways*. RNASE2* had 166 co-expressed genes, while *CASP5* had *346* including *MYBL1*, of which 163 were overlapped, containing *HAVCR2* and *TDO2* (**Figures 7G, H**). These genes were chiefly gathered in “inflammatory response”, “regulation of leukocyte activation”, “cellular response to cytokine stimulus”, etc. (**Figure 7I**), implying that RNASE2 and CASP5 may interact with HAVCR2 and TDO2 to facilitate ATC *via* immune evading and immunosuppression, and *CASP5* might be regulated by MYBL1.

## Discussion

Cancer is an extremely heterogeneous disease, which must be manipulated by specific drivers in various types of cancer. ATC is a kind of thyroid cancer with much more aggressive behaviors than other thyroid cancer, like PTC, FTC, and PDTC (6, 7). Nevertheless, the underlying mechanisms and key driver molecules promoting ATC traits remain unknown. We assumed that some hallmarks not just one must be responsible for the overgrowth and progression of ATC within a few months. Up to now, a total of 14 hallmarks of cancer have been concluded. They are 14 biological capabilities acquired during the multistep development of human carcinoma, including sustaining proliferative signaling, evading growth suppressors, avoiding immune destruction, enabling replicative immortality, tumor-promoting inflammation, activating invasion & metastasis, inducing or accessing vasculature, genome instability & mutation, resisting cell death, deregulating cellular metabolism, unlocking phenotypic plasticity, nonmutational epigenetic reprogramming, polymorphic microbiomes, and senescent cells (51, 52). These hallmarks may provide reasons for the biological behavior of tumors. In this study, we attempted to provide new insights into ATC-specific tumor biology explored by WGCNA.

WGCNA can detect modules that relate to external clinical traits, and these modules can reflect true biological signals, like pathways (36). Therefore, we identified ATC-specific modules through WGCNA and then investigated ATC-specific functions and pathways enriched by hub genes of these modules instead of by DEGs in ATC compared to normal thyroid samples or other thyroid cancers like previous studies. As such, the dominant perturbations contributing to ATC-specific tumor biology could be revealed, as well as the most potential driver genes. As expected, the three most potential ATC-specific modules were detected and colored by turquoise, magenta, and blue. With enrichment analysis, we revealed that hub genes in the turquoise module were mainly enriched in mitotic cell cycle, tube morphogenesis, cell differentiation, and cell-cell adhesion, and hub genes in the magenta module were mainly clustered in the extracellular matrix organization, blood vessel development, positive regulation of cell motility, and regulation of Wnt signaling pathway, while hub genes in the blue module mainly participated in the inflammatory response, innate immune response, and adaptive immune response (**Figures 2A–C**). Dysregulations of these biological processes and pathways can facilitate ATC growth and metastasis. For instance, up-regulated mitogenic signaling will accelerate the cell growth-and-division cycle and thus make the tumor large fast, undifferentiated cells have pliability in cell state, which can enhance multiple aspects of tumor progression (53), and the vasculature of invasive tumor is continually sprouting new vessels to sustain expand neoplastic growth, while extracellular matrix organization alteration (54), Wnt signaling pathway activation, and cancer immunoediting will promote tumor metastasis (51, 52, 55). Therefore, these findings may provide clues to elucidate molecular mechanisms underlying ATC aggressive behaviors and might make it possible to offer novel early diagnostic biomarkers and multiple therapeutic targets for ATC patients with more specificity.

In addition, we noticed that hub genes of the blue module were mainly located on the external side of the plasma membrane and were involved in immunosuppression. Theoretically, molecules on the outside of the cell membrane are easier to be identified and targeted, which will facilitate the development of new treatment targets for ATC patients in the clinic. While immunosuppression is a key factor in tumor progression. Our study may lay a foundation for further exploring the immunosuppression mechanism of ATC and provide novel targets for immunotherapy. Apart from those, we also noted that there were hub genes regulated by TP53 in these three ATC-specific modules. Furthermore, based on the cBioPortal database, we discerned that much more *TP53* mutation existed in ATC than in other thyroid cancers consistent with several previous studies (3, 14, 16, 56), and patients with *TP53* mutation had poorer overall survival, suggesting that *TP53* mutation may play an important role in occurrence and development of ATC. The current study might provide novel ATC-specific genes involved in the TP53 signaling pathway.

After confirming true biological signals could be reflected by these three ATC-specific modules, from which, we further screened out 9 top genes and 8 TFs with the highest aGS for ATC, as well as 4 ICGs, of which 18 genes (*ADAM12, TWIST1, SRPX2, RNASE2, SIRPB2, CASP5, IL10, KIAA1524, SNAI2, ELF4, IFI16, MYBL1, SHOX2, E2F7*, *CD86, CD274, HAVCR2*, and *TDO2)* were up-regulated in ATC, while 2 genes (*FOXE1* and *TSHR*) were down-regulated, and all of them were specific to ATC and had diagnostic values for ATC patients. To our knowledge, 5 genes *TWIST1*, *SNAI2*, *TSHR*, *FOXE1*, and *CD274* (PD-L1) have been reported to be differentially expressed in ATC compared to normal thyroid samples and other thyroid cancer samples and one gene (*E2F7*) has been identified in ATC but without confirmation in clinical samples (20) or without comparisons to other thyroid carcinomas (57). Paolo Salerno et al. revealed that TWIST1 was up-regulated in ATC compared to normal thyroids, WDTC and PDTC, and could promote cell migration, invasion, and resistance to apoptosis (58). It is widely accepted that SNAI2 is one effector of epithelial-mesenchymal transition and a repressor of E-cadherin (59). One research revealed that SNAI2 was more expressed in ATC than in normal tissues and other thyroid cancers (60). Very little expression of TSHR and FOXE1 are detected in ATC and their expression levels have positive correlations with the degree of differentiation (50, 61). PD-L1, which is a promising treatment target for ATC patients recently (12) but is positively expressed in less than 30% of ATC samples (46, 62) and thus more potential effective targets are needed to be developed for ATC.

Therefore, we validated the remaining 15 novel ATC-specific genes including *E2F7* with our clinical samples (12 paired normal and PTC tissue samples and one ATC sample) and demonstrated that 8 out of them (*ADAM12, RNASE2, CASP5, KIAA1524, E2F7, MYBL1, SRPX2*, and *HAVCR2*) were highly expressed in this one ATC sample compared to PTC and paired normal thyroid samples at mRNA expression level. Unfortunately, with our limited protein samples, we showed that only one protein ADAM12 had higher expression in one ATC sample relative to PTC and paired normal thyroid samples. Notably, we found that tightly co-expressed genes of validated top key genes mainly enriched in mitotic cell cycle, regulation of Wnt signaling pathway, extracellular matrix organization, regulation of angiogenesis, positive regulation of cell motility, cell differentiation, inflammatory response, adaptive immune system and so on, suggesting that these key genes were likely to contribute to ATC through these biological processes or pathways and play pivotal roles in them, and thus might be promising diagnostic and therapeutic targets for ATC patients in the clinic. ADAM12 could promote cell invasion, metastasis, and apoptosis (63, 64). Of note, our study showed that *ADAM12* was a co-expressed gene of *TWIST1* and Mark A. Eckert et al. elucidated that ADAM12 regulated by TWIST1 could enhance tumor invasion and metastasis through invadopodia and focal adhesion in breast cancer cells (65), indicating that their regulatory relationship might exist in ATC cells. Another study revealed that LINC00284 might promote the progression of thyroid cancer by competitively binding to miR-30d-5p and thus activating ADAM12-dependent Notch signaling pathway (66). A recent study reported that E2F7 could enhance PTC cell growth (67). Tingting Chao and co-workers revealed that KIAA1524, also known as CIP2A, was more expressed in PTC than in thyroid non-tumor tissues and benign tumor tissues (68). A large cohort study discovered that HAVCR2 (TIM-3) was positively expressed in 48% medullary thyroid carcinoma (69).

The present study has some limitations. First, we selected and downloaded six datasets of thyroid cancer from GEO database. Due to integrating different dataset, we employed PCA and hierarchical cluster to ensure the availability of data for WGCNA and removed batch effects for DEG analysis in addition to raw data quality control and preprocessing. These measures to some extent guarantee the reliability of our research results. However, our validated samples are limited, especially ATC sample, just one. Therefore, more clinical samples are essentially needed to prove our findings. Second, our study provided three ATC-specific modules and revealed biological signals involved in, all hub genes of which may contribute to ATC. However, we just chose top 3 hub genes and TFs with highest aGS and currently potential ICGs from three ATC-specific modules for further validation. Helpfully, we listed all hub genes in tables behind biological signals enriched, which may offer a clue for other researchers to further investigations. Third, for key novel ATC-specific genes screened in this study, we performed preliminary verification, *in vivo* and *in vitro* experiments will be certainly indispensable to uncover the molecular mechanisms underlying aggressive phenotypes of ATC and further clinical trials must be carried out.

In conclusion, by employing WGCNA on a gene expression matrix covering normal thyroid samples and other thyroid cancer (PTC, FTC, and PDTC) samples, we detected three most potential ATC-specific modules, based on which, we revealed biological signals and pathways altered in ATC and validated 8 novel ATC-specific genes in addition to confirming existed ones. This study may throw a light on molecular mechanisms underlying aggressive phenotypes of ATC and provide novel diagnostic biomarkers and therapeutic targets for ATC patients.

## Data availability statement

The datasets presented in this study can be found in online repositories. The names of the repository/repositories and accession number(s) can be found in the article/**Supplementary Material**.

## Ethics statement

The studies involving human participants were reviewed and approved by the Ethics Committee of Zhongnan Hospital of Wuhan University. The patients/participants provided their written informed consent to participate in this study.

## Author contributions

XD and GW conceived and designed the study. XD, QY, and CL analyzed the data. XD, JH, and JL collected clinical samples. XD, YY, and GX carried out experiments. XD drafted the manuscript. GW, YY, and WC revised the paper. All authors contributed to the article and approved the submitted version.

## Acknowledgments

The authors appreciate GEO database and the researchers sharing the original data.

## Conflict of interest

The authors declare that the research was conducted in the absence of any commercial or financial relationships that could be construed as a potential conflict of interest.

## Publisher’s note

All claims expressed in this article are solely those of the authors and do not necessarily represent those of their affiliated organizations, or those of the publisher, the editors and the reviewers. Any product that may be evaluated in this article, or claim that may be made by its manufacturer, is not guaranteed or endorsed by the publisher.

## Abbreviations

ATC, Anaplastic thyroid carcinoma; WGCNA, weighted gene co-expression network analysis; TF, transcription factor; ICG, immune checkpoint gene; WDTC, well-differentiated thyroid cancer; PTC, papillary thyroid carcinoma; FTC, follicular thyroid carcinoma; PDTC, poor-differentiated thyroid carcinoma; OS, overall survival; DEG, differentially expressed gene; GEO, Gene Expression Omnibus; NUSE, normalized unscaled standard error; KNN, K Nearest Neighbors; PCA, principal component analysis; ME, module eigengene; aGS, absolute gene significance; MM, module membership; BP, biological process; GO, Gene Ontology; KEGG, Kyoto Encyclopedia of Genes and Genomes; MF, molecular function; CC, cellular component; TRRUST, Transcriptional Regulatory Relationships Unraveled by Sentence-based Text; ROC, receiver operating characteristic; AUC, area under the curve; ANOVA, one-way analysis of variance; IHC, immunohistochemistry.
