# Supplementary material for: Weighted gene co-expression network reveals driver genes contributing to phenotypes of anaplastic thyroid carcinoma and immune checkpoint identification for therapeutic targets
Source: Front Oncol. 2022 Dec 1;12:1018479. doi: 10.3389/fonc.2022.1018479 (PMC9751455; doi:10.3389/fonc.2022.1018479)
Supplement: Supplementary file 11 [file DataSheet_1.pdf]

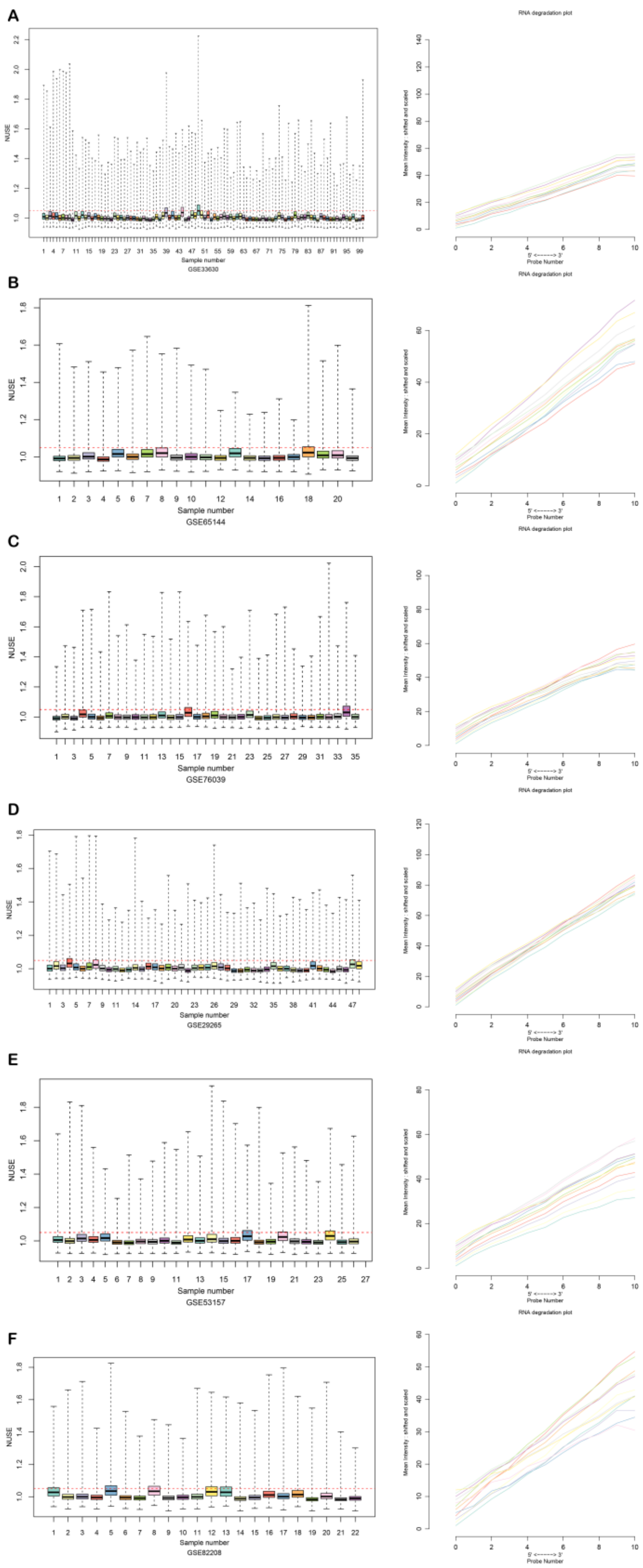

Supplementary Figure 1. Raw data quality control at the probe level. (**A-F left**) Normalized unscaled standard error (NUSE) boxplots for every dataset, each box of which represents a gene chip or sample. The red dotted line denoted the alarming value of 1.05. (**A-F right**) The corresponding RNA degradation plots of the same datasets with the left, each line represents one sample.

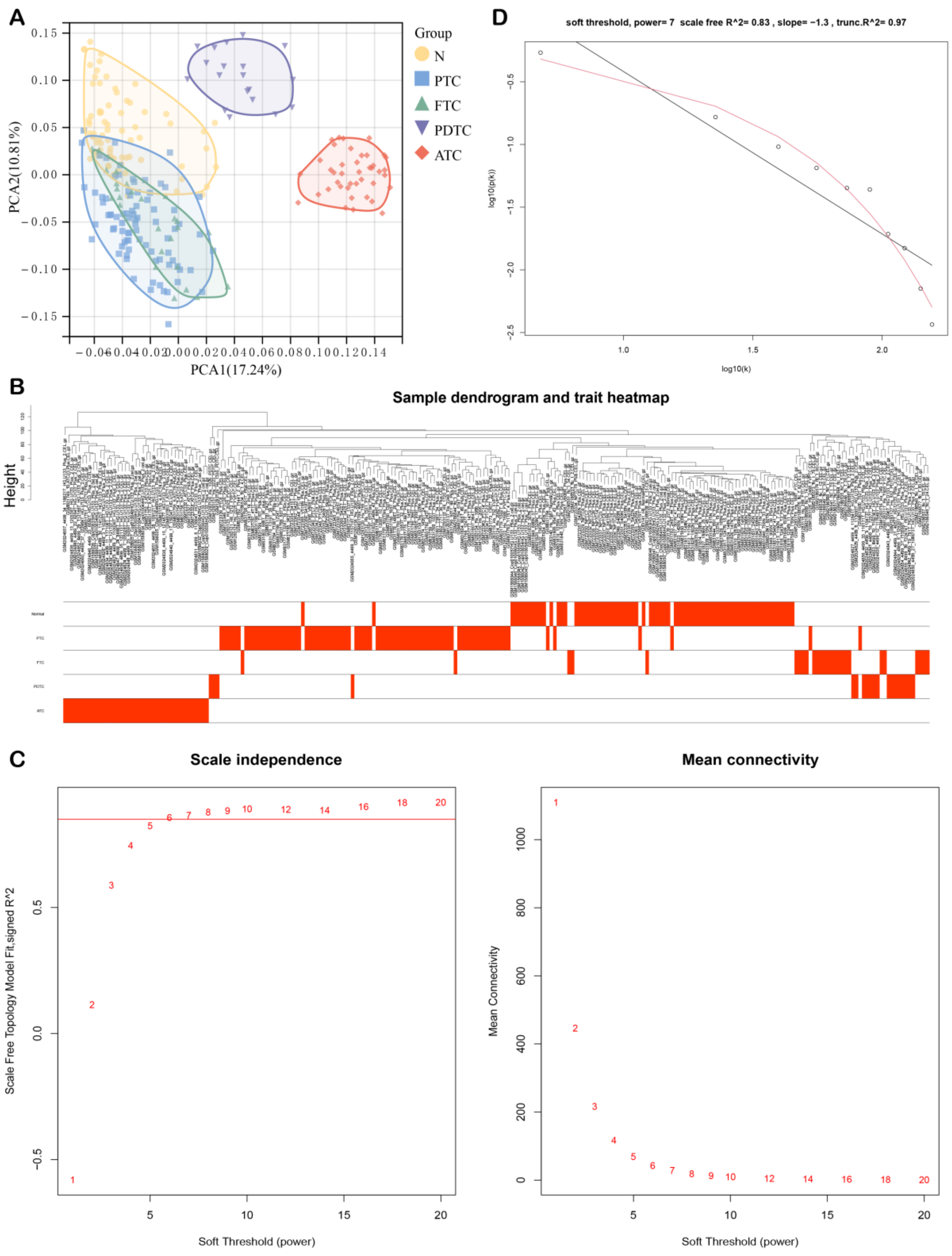

Supplementary Figure 2. Sample clustering and soft threshold selection. (**A**) PCA plot of all samples used for WGCNA. (**B**) Hierarchical clustering dendrogram of all samples. (**C**) The corresponding signed R^2 (scale-free topology fit) of different soft thresholds (powers). The red line represents signed R^2 = 0.85 and a network with signed R^2 > 0.8 could be considered to meet scale-free topology (left). The mean connectivity under different soft thresholds (right). (**D**) Scale-free topology plot of the network that was built with the soft threshold = 7. The scale-free R^2 = 0.83 indicates that the network satisfied the scale-free topology.

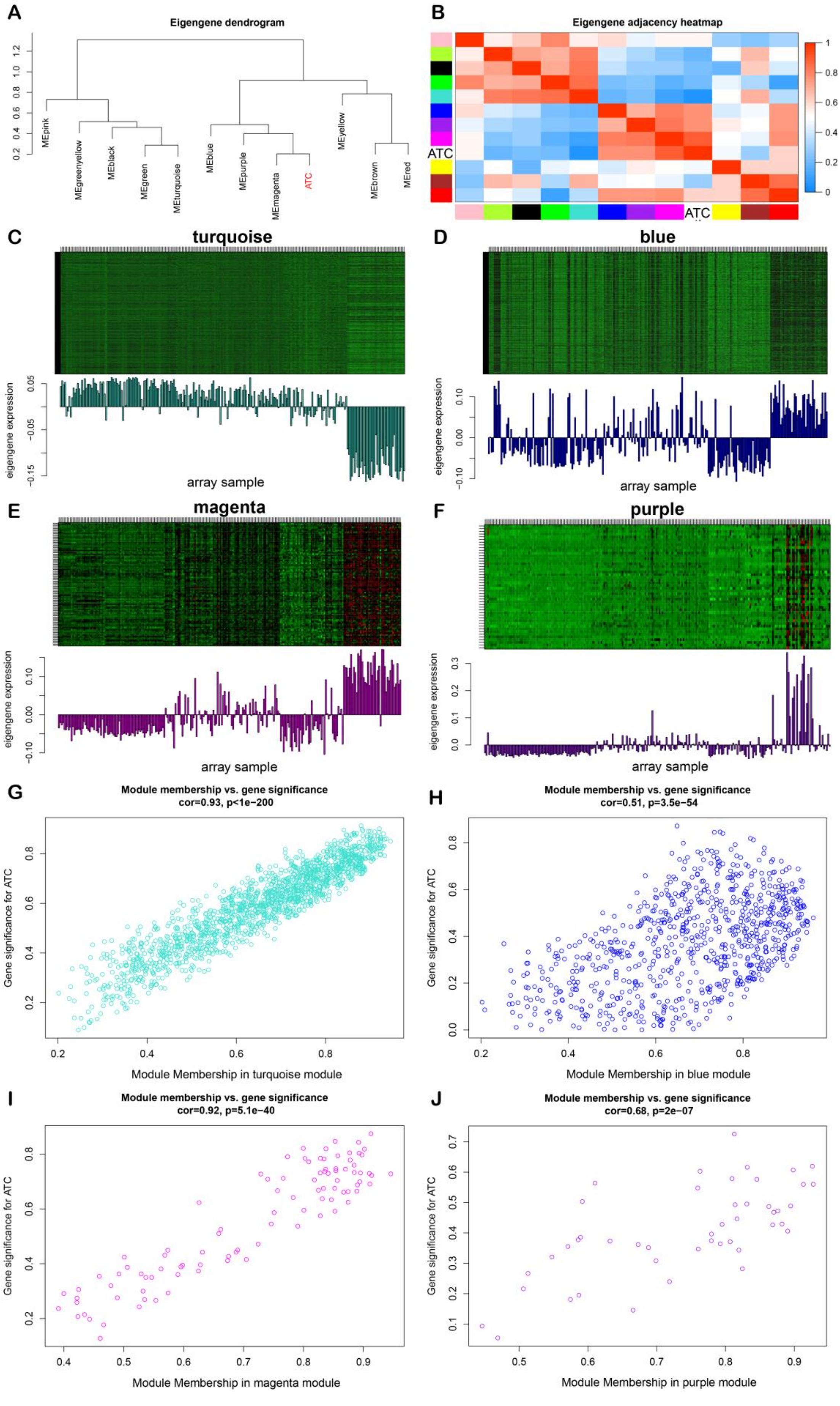
A
Eigengene dendrogram
MEpink
MEgreenyellow
MEblack
MEgreen
MEturquoise
MEblue
MEpurple
MEmagenta
ATC
MEyellow
MEbrown
MEred
B
Eigengene adjacency heatmap
ATC
C
turquoise
eigengene expression
array sample
D
blue
eigengene expression
array sample
E
magenta
eigengene expression
array sample
F
purple
eigengene expression
array sample
G
Module membership vs. gene significance
cor=0.93, p<1e−200
Gene significance for ATC
Module Membership in turquoise module
H
Module membership vs. gene significance
cor=0.51, p=3.5e−54
Gene significance for ATC
Module Membership in blue module
I
Module membership vs. gene significance
cor=0.92, p=5.1e−40
Gene significance for ATC
Module Membership in magenta module
J
Module membership vs. gene significance
cor=0.68, p=2e−07
Gene significance for ATC
Module Membership in purple module

Supplementary Figure 3. The associations among modules, ME expression across all samples and gene significance for ATC. **(A)** Hierarchical clustering of MEs of every module and ATC. Branches of dendrogram group together eigengenes that are positively correlated. **(B)** Heatmap plot of the adjacencies in the eigengene network including ATC. Each row and column correspond to one ME (labeled by color) or ATC. In the heatmap, the blue color denotes low adjacency (negative correlation), while red denotes high adjacency (positive correlation). **(C-F)** ATC-specific module eigengene expression across all samples. They are normal thyroid tissue samples, PTC, FTC, PDTC, and ATC samples in the sequence (x-axis). Red corresponds to high- and green to low expression values. **(G-J)** Scatterplot of GS (y-axis) vs. module membership (x-axis) in four ATC-specific modules. In these modules, genes with high module membership also have high GS. A bubble represents a gene, and the color denotes the corresponding module.

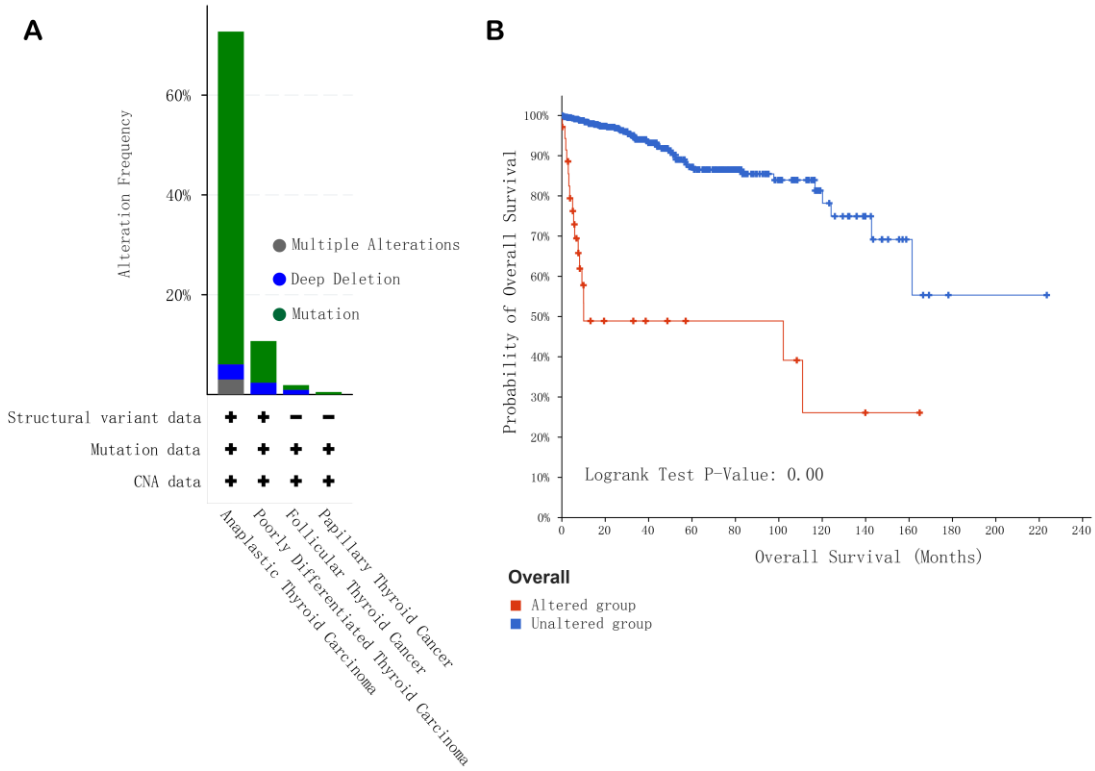

Supplementary Figure 4. Genetic alteration and corresponding survival analysis of TP53 in thyroid cancers performed by cBioPortal tool.

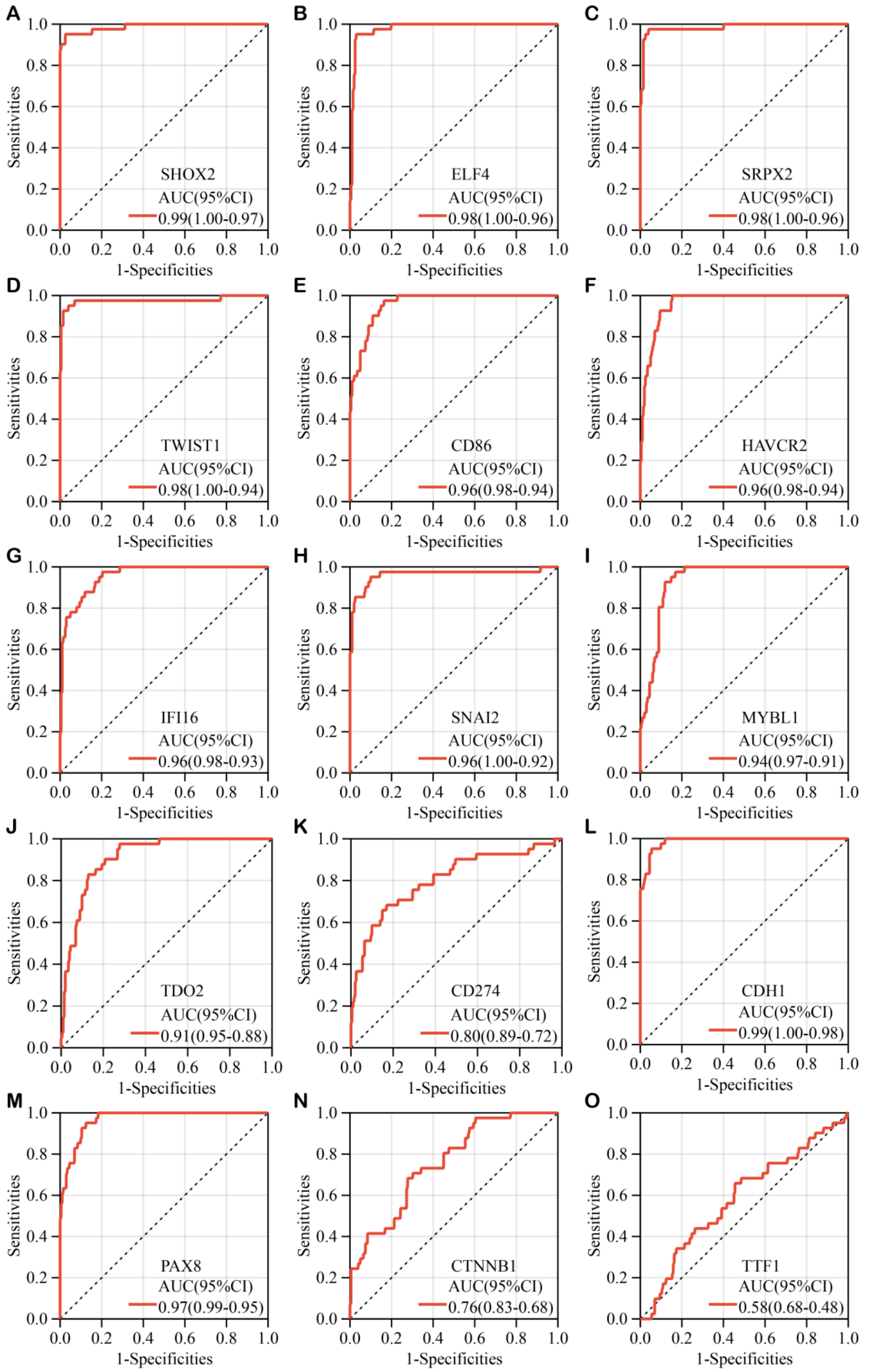

Supplementary Figure 5. Diagnostic ROC analysis of the rest of hub genes and four existed ATC markers.

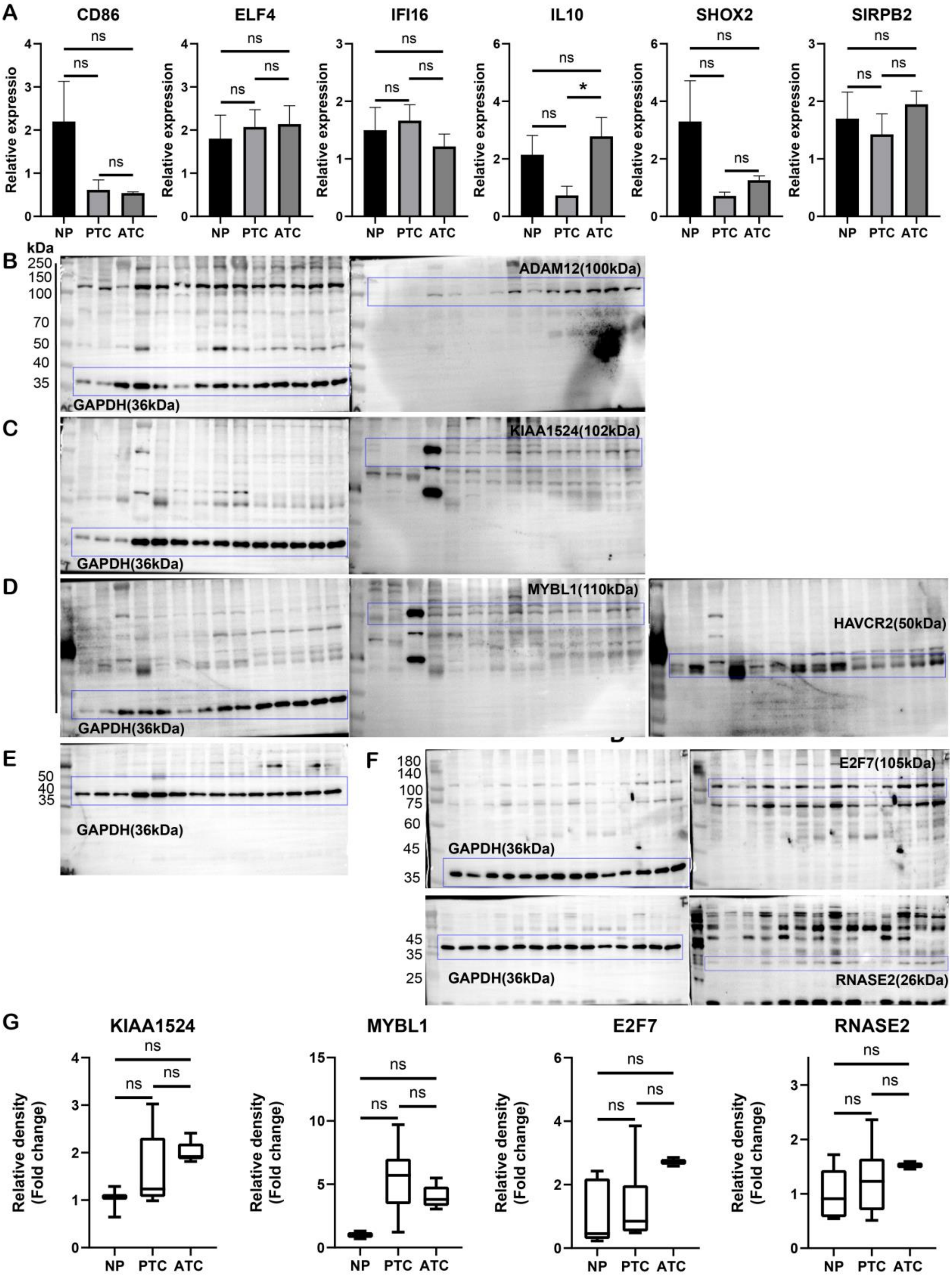

Supplementary Figure 6. Validation of novel ATC-specific key genes. **(A)** The mRNA expression levels of 6 genes without significant changes in one ATC sample compared to PTC and paired normal thyroid samples evaluated by qRT-PCR. **(B)** The expression levels of ADAM12 and GAPDH in the same western blot detected successively. The loading samples from left to right are

molecular weight mark, 3 normal thyroid tissue samples, 6 PTC samples, and five replicates of one ATC samples repeated twice paired PTC number 1-6 (and PTC samples), and. The bands framed by light blue rectangle are target bands, the corresponding observed molecular weights of which (provided by primary antibody company) are shown in parentheses. **(C)** The same as B, but KIAA1524 and GAPDH in turn. **(D)** The same as B, but MYBL1, HAVCR2, and GAPDH in turn. **(E)** The same as B, but only GAPDH. **(F)** The whole WB membranes of E2F7 and RNASE2, and the corresponding GAPDH. The loading samples from left to right are molecular weight mark, 6 paired PTC samples (normal thyroid tissue and PTC samples), and one ATC samples repeated twice. **(G)** Semi-quantitative analysis of proteins according to the gray density of their bands. *p < 0.05. ns, no significance; NP, PTC paired normal thyroid tissues.

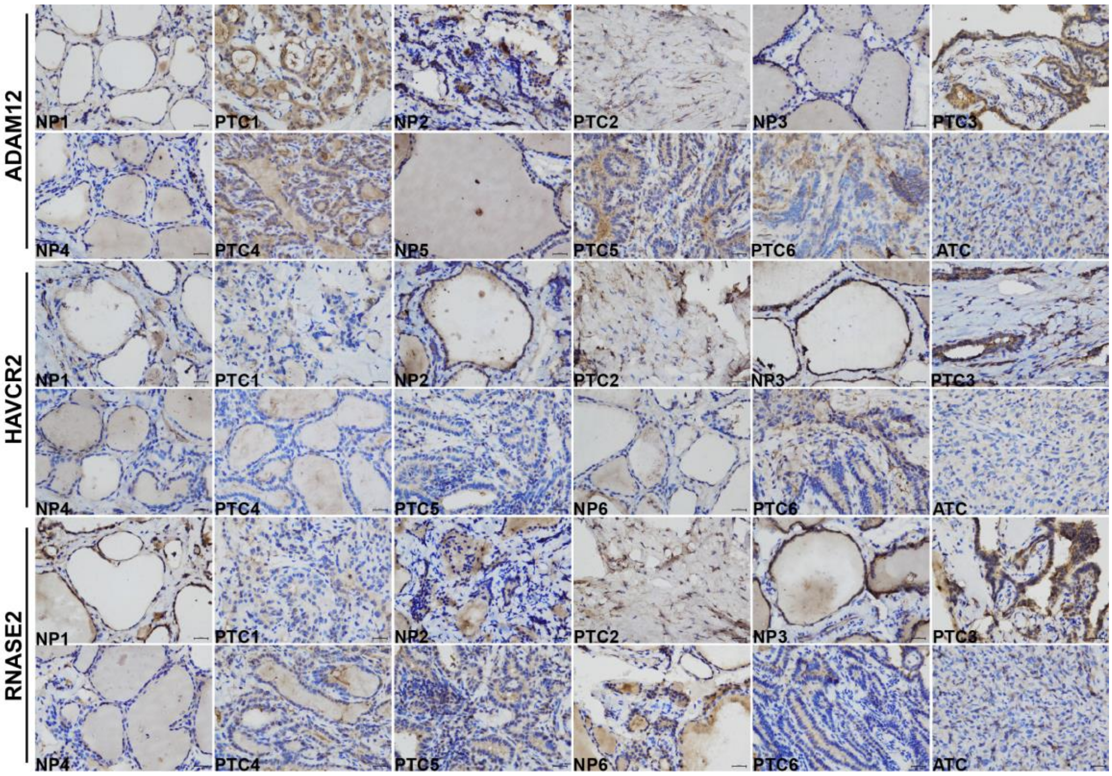

Supplementary Figure 7. Protein expression levels of ADAM12, HAVCR2, and RNASE2 in clinical samples detected by immunohistochemistry (IHC) staining. NP, PTC paired normal thyroid tissues.

Supplementary Table 1. Primer sequences of key genes used for qRT-PCR in the present study

Candidate gene	Primer Sequence
ADAM12	Forward 5’-CGCTCGAAATTACACGGGTC-3’<br>Reverse 5’-AGTCCCCTGAGACCAGAACA-3’
SRPX2	Forward 5’-AGGAAACTTTTGGCAGATGTGG-3’<br>Reverse 5’-CTAGAACGAGTGGCTCCTGG-3’
RNASE2	Forward 5’-GAGATCAACGACGAGACCCTC-3’<br>Reverse 5’-GAGGAGTGCTGATACAGGAGC-3’
SIRPB2	Forward 5’-GTGAGCACTCAGGACCAACA-3’<br>Reverse 5’-TGTGCTCCCTGGTGACATTG-3’
CASP5	Forward 5’-GTTAGCTATGGCTGAAGACAGT-3’<br>Reverse 5’-TTGTCCAGCCACGTTGTTCTT-3’
IL10	Forward 5’-GCTCAGCACTGCTCTGTTGC-3’<br>Reverse 5’-TTCACTCTGCTGAAGGCATCTC-3’
TSHR	Forward 5’-AGAGCTGAGAATGAGGCGAT-3’<br>Reverse 5’-CAGGGTGGAGACGAACACC-3’
CIP2A (KIAA1524)	Forward 5’-GACTCCACTGCCTGCTTGAA-3’<br>Reverse 5’-AAATTACCTCCAAGTGCCGC-3’
HAVCR2	Forward 5’-CTTACCACCAGGGGACATGG-3’<br>Reverse 5’-TGGCCAATCTAGAGTCCCGT-3’
TDO2	Forward 5’-GCAAATCCTCTGGGAGTTGGA-3’<br>Reverse 5’-TCGGTGCATCCGAGAAACAA-3’
CD86	Forward 5’-CAAGCAAGAGCACTGTCCCT-3’<br>Reverse 5’-AGCTCCCCGTACCTCCTAAG-3’
E2F7	Forward 5’-CTGCTGCGCTAGACTTGGATA-3’<br>Reverse 5’-CACAGCCTCTGTGACAACTCA-3’
FOXE1	Forward 5’-CGCTGTTCTAGGGAACGGAA-3’<br>Reverse 5’-CTGGGCAGTAACAGCCATGA-3’
ELF4	Forward 5’-TGAAAACTGGTTTCTGCCACC-3’<br>Reverse 5’-GGGCCCGAGAACTGTGAAGA-3’
IFI16	Forward 5’-AGAATAGGAGCAAGCCAGCA-3’<br>Reverse 5’-AGAAACGGAACCGCAGGATG-3’
SHOX2	Forward 5’-AGTACAAAATGTGTCGCCTCCA-3’<br>Reverse 5’-AAAGATGACTCGAACCCCATCA-3’
MYBL1	Forward 5’-GAGGGAAGCGGCTAGAGGAT-3’<br>Reverse 5’-AAGTACCGCATAGGAGCAGG-3’
GAPDH	Forward 5’-GACAGTCAGCCGCATCTTCT-3’<br>Reverse 5’-GCGCCCAATACGACCAAATC-3’

Supplementary Table 2. The dilutions of antibodies used in this study for western blotting

Gene symbol	Primary antibody	Secondary antibody
ADAM12	1/1000	1/8000
RNASE2	1/1500	1/8000
CASP5	1/1000	1/8000
KIAA1524	1/3000	1/8000
HAVCR2	1/1000	1/8000
TDO2	1/1000	1/8000
E2F7	1/1000	1/8000
FOXE1	1/2000	1/8000
MYBL1	1/1000	1/8000
GAPDH	1/20000	1/10000

Supplementary Table 7. Enrichment results of hub genes of ATC-specific modules based on other databases investigated by the Matascape web.

Module	Database	Description	LogP	Count	Gene symbol
Blue	DisGeNET	Immunosuppression	-54	96	ALOX5|AREG|BTK|CD1D|CD247|CD14|CD86|CD33|CD38|CD69|CD72|CD52|CCR5|CCR7|CSF1R|CYBB|DDIT3|FCGR2B|GEM|GZMB|HLA-C|HLA-DPB1|HLA-DQA1|HLA-DQB1|HLA-DRA|HLA-DRB1|HLA-E|HLA-G|HMOX1|ICAM3|IFI16|IFIT2|IFNG|IL1B|IL2RA|IL2RG|IL4R|IL6|IL7|CXCR2|IL15|IL15RA|IL18|IDO1|CXCL10|ITGA4|ITGAL|ITGAM|ITGB2|LAG3|LGALS9|LYZ|MMP9|MRC1|MYC|MYD88|P2RX7|CFP|PIK3CG|PRF1|PRKCB|PTAFR|PTGS2|S100A9|S100B|CCL2|CCL5|CCL18|CCL22|SPP1|TAP1|TDO2|TIMP2|TLR2|TLR4|VAV1|VCAM1|CXCR4|TNFSF11|TNFSF10|IL18R1|AIM2|EBI3|LILRB2|TNFSF13B|PIM2|PTPN22|CD274|ICOS|TRAT1|SLAMF8|CLEC7A|HAVCR2|TIGIT|IL4I1|CCR2
	PaGenBase	Tissue-specific: spleen	-1.00E+02	131	AOAH|RHOH|BTK|C1QA|C1QB|C1QC|CASP5|RUNX3|CD3D|CD3E|CD3G|CD37|CD48|CD53|CD72|CCR1|CCR6|CCR7|CR1|DOCK2|TYMP|ADGRE1|EVI2B|FCGR3B|FYB1|GZMH|GZMB|GZMK|HCK|HCLS1|HLA-DQB1|HMOX1|IRF8|IFI35|IL2RA|IL2RB|IL2RG|CXCR2|IL10RA|ITGA4|ITGAL|ITK|KLRD1|LAG3|LSP1|LTB|LY9|CD180|LYN|SH2D1A|MMP9|MMP12|MYO1F|NFKBIE|NKG7|P2RX5|P2RY6|CFP|PIK3CG|PRF1|PTPRC|PTPRCAP|RAC2|RNASE6|CCL5|XCL1|SELL|SIPA1|SLA|TBXAS1|TLR2|TLR4|TNFRSF1B|VAV1|VCAM1|CXCR4|LST1|EOMES|MAD1L1|GPR65|CST7|IL18R1|HCAR3|MAP3K14|DOK2|STK17B|AIM2|THEMIS2|SCO2|NR1H3|IGSF6|LILRB2|BATF|CXCL13|GNLY|LILRB5|LILRA3|PIM2|CORO1A|SLCO2B1|CD300A|DAPP1|GPR171|ADGRE2|IL21R|TLR8|P2RY13|SASH3|LAX1|SLAMF7|GPSM3|RGS18|LILRA6|TNFAIP8L2|DOK3|TRAF3IP3|DOCK8|RASSF5|SIGLEC10|KLHL6|ANKRD22|CD300LF|PYHIN1|BTLA|MCEMP1|MPEG1|NAPSB|ARHGAP30|ZC3H12D|CALHM6|NCF1
	PaGenBase	Tissue-specific: Blood	-1.00E+02	102	AIF1|AOAH|AQP9|BCL2A1|C5AR1|CASP1|CASP4|CCR1|CSF2RB|CSF3R|CTSS|CYBB|EVI2B|FCER1G|FCGR2A|FCGR3B|FCN1|FGR|FPR1|FYB1|GBP2|GZMH|GZMA|GZMB|HCK|HCLS1|HSPA6|ICAM3|IFIT2|IL6R|CXCR2|IL10RA|IRF1|ITGB2|LCP1|LCP2|LSP1|LYN|MNDA|MYO1F|MX2|MYD88|NCF2|NCF4|NKG7|CFP|PLEK|PSMB9|PTPRC|RGS2|RNASE6|S100A4|CCL5|SELL|SELPLG|STK10|TLR1|TLR2|TNFRSF1B|TYROBP|LAPTM5|LST1|TNFSF10|HCAR3|VNN2|PSTPIP1|NMI|THEMIS2|GMFG|CYTIP|CLEC2B|IGSF6|LILRB2|GNLY|LILRA3|CORO1A|CD300A|LY96|PILRA|ADGRE2|CLEC4A|CKLF|TLR8|CRLF3|P2RY13|TMEM140|GPSM3|NOD2|MS4A6A|CLEC7A|LILRA6|PLBD1|NPL|RASSF5|FERMT3|GBP5|NIBAN1|TAGAP|JAML|C1orf162|ARHGAP30|CCR2
	PaGenBase	Tissue-specific: thymus	-34	47	RHOH|CD1D|CD2|CD3D|CD3E|CD3G|CD247|CDKN2C|CCR6|DUSP2|ETS1|FYB1|IRF8|IL7R|ITGA4|ITK|LAIR1|LCK|LTB|CD180|SH2D1A|CXCL9|MMP12|OAS1|PTPRC|PTPRCAP|CCL13|CCL18|CCL22|XCL1|ZAP70|ST8SIA4|STK17A|CD163|CYTIP|SEPTIN6|PTPN22|ADAMDEC1|TRBC1|TRAC|ICOS|GPR171|TRAT1|XAF1|BCL11B|PVRIG|TRAF3IP3
	TRRUST	Regulated by: TP53	-4.7	14	ALOX5|CASP1|DNMT1|ELF4|IFI16|MYC|PLAGL1|PMAIP1|S100B|TBXAS1|TYMS|MAD1L1|TNFRSF10A|GPNMB
Turquoise	TRRUST	Regulated by: TP53	-7.9	29	BIRC5|ATF3|CCNA2|CCNB1|CDK1|CHEK1|CRYAB|DUSP1|EGR1|EZH2|FOXM1|ID1|ID2|IGF1R|CD82|MKI67|MMP1|NME1|PTPN13|THBS1|VEGFA|BTG2|CCNA1|PRC1|CARM1|DKK1|UHRF1|RRM2B|E2F7
Magenta	TRRUST	Regulated by: TP53	-2.7	4	VCAN|CTNNB1|PDGFRB|TP53I3
Blue	TRRUST	Regulated by: TP53	-4.7	14	ALOX5|CASP1|DNMT1|ELF4|IFI16|MYC|PLAGL1|PMAIP1|S100B|TBXAS1|TYMS|MAD1L1|TNFRSF10A|GPNMB

Supplementary Table 11. TFs identified from hub genes of turquoise, magenta, and blue module and ICGs explored from hub genes of blue module

Module	TF	ICG
Turquoise	*BTG2, TRIB3, HOXA1, PIR, NFIA, HES1, ISL1,* ***FOXE1****, FOXM1, ID1, NFIL3, HLF, PBX1, PITX1, IRF7, TCF19, HOXA5, E2F7, NKX2-1, TEAD4, FOXG1, NR3C2, CRABP2, HHEX, PAX6, SPI1, HOXC10, GLIS3, KLF5, CBX7, ID2, TWIST2, GTF2I, RORC, FOSB, SHOX2, HOXB7, BHLHE41, SATB1, KAT2B, HOPX, E2F8, MSC, SOX4, ATF3, EZH2, FOXC1, MAL, IRF6, MYBL2, EGR1, ESRRG, ARNTL2, CDCA7L, EGR2, FHL2, ZBTB16, MLLT3, ID4, BRIP1, FOS,* ***PAX8****, CEBPA, SOX11, HOXC6, UHRF1, HMGA1, AR, FOSL1, TOB1*	
Magenta	***CTNNB1,*** *HEY1, TWIST1, SOX17, SNAI2*	
Blue	*RUNX3, IFI16, ETV7, ELF4, MCM5, EOMES, MYC, TNFAIP3, MYBL1, GATA3, RELB, BACH2, MAFF, IRF1 NR2F1, NR1H3, ZBTB2, PLAGL1, KLF6, BATF, DNMT1, DDIT3, ZNF300, ETS1, GATA5, IRF8*	*IDO1, BTLA, LGALS9, LAG3, CD48, TIGIT, ICOS, TDO2, HAVCR2, CD86,* ***CD274***

The genes in bold have been widely accepted in ATC. TF, transcription factor; ICG, immune checkpoint gene.

Supplementary Table 12. Expression changes of key genes in ATC compared to normal and PTC samples

			ATC vs. N		ATC vs. PTC	
		Gene symbol	Log_2FC	adj.P.Val	Log_2FC	adj.P.Val
Magenta module		*ADAM12*	3.22	1.86E-50	2.44	1.51E-29
		SRPX2	2.62	6.96E-51	2.08	1.10E-34
	TFs	*TWIST1*	4.05	2.75E-43	4.15	6.22E-38
		SNAI2	3.25	1.10E-29	3.23	9.74E-28
Turquoise module		*IL10*	2.09	1.67E-38	1.89	6.30E-38
		TSHR	-5.57	3.29E-31	-5.23	6.52E-28
		KIAA1524	2.20	8.36E-34	2.52	2.89E-37
	TFs	*E2F7*	2.61	3.71E-33	2.45	2.62E-29
		FOXE1	-2.38	1.43E-27	-1.95	7.32E-22
		SHOX2	2.31	9.05E-33	2.27	1.87E-30
Blue module		*RNASE2*	3.17	3.52E-38	2.70	2.60E-29
		SIRPB2	2.44	5.62E-40	1.80	5.87E-29
		CASP5	1.53	3.47E-27	1.43	2.89E-28
	TFs	*IFI16*	1.99	9.77E-24	1.31	1.96E-15
		ELF4	2.30	6.15E-31	1.78	1.32E-26
		MYBL1	1.87	5.84E-18	2.06	1.16E-28
	ICGs	*TDO2*	2.37	3.82E-26	1.58	6.70E-15
		HAVCR2	1.64	1.78E-26	1.47	4.59E-28
		CD86	2.51	1.54E-23	1.81	1.15E-17
		CD274	1.67	1.84E-15	1.32	2.18E-09

Adj.P.Val, adjust p value.
